# Reproductive Regulation of PrRPs in Teleost: The Link Between Feeding and Reproduction

**DOI:** 10.3389/fendo.2021.762826

**Published:** 2021-11-03

**Authors:** Chuanhui Xia, Xiangfeng Qin, Lingling Zhou, Xuetao Shi, Tianyi Cai, Yunyi Xie, Wei Li, Ruixin Du, Yu OuYang, Zhan Yin, Guangfu Hu

**Affiliations:** ^1^ College of Fisheries, Hubei Province Engineering Laboratory for Pond Aquaculture, Huazhong Agricultural University, Wuhan, China; ^2^ State Key Laboratory of Freshwater Ecology and Biotechnology, Institute of Hydrobiology, Chinese Academy of Sciences, Wuhan, China

**Keywords:** reproduction, food intake, PrRP, grass carp, luteinizing hormone

## Abstract

Prolactin-releasing peptide (PrRP), a sort of vital hypothalamic neuropeptide, has been found to exert an enormous function on the food intake of mammals. However, little is known about the functional role of PrRP in teleost. In the present study, two PrRP isoforms and four PrRP receptors were isolated from grass carp. Ligand-receptor selectivity displayed that PrRP1 preferentially binds with PrRP-R1a and PrRP-R1b, while PrRP-R2a and PrRP-R2b were special receptors for PrRP2. Tissue distribution indicated that both PrRPs and PrRP-Rs were highly expressed in the hypothalamus-pituitary-gonad axis and intestine, suggesting a latent function on food intake and reproduction. Using grass carp as a model, we found that food intake could significantly induce hypothalamus PrRP mRNA expression, which suggested that PrRP should be also an anorexigenic peptide in teleost. Interestingly, intraperitoneal (IP) injection of PrRPs could significantly induce serum luteinizing hormone (LH) secretion and pituitary LHβ and GtHα mRNA expression in grass carp. Moreover, using primary culture grass carp pituitary cells as a model, we further found that PrRPs could directly induce pituitary LH secretion and synthesis mediated by AC/PKA, PLC/IP3/PKC, and Ca2+/CaM/CaMK-II pathways. Finally, estrogen treatment of prepubertal fish elicited increases in PrRPs and PrPR receptors expression in primary cultured grass carp hypothalamus cells, which further confirmed that the PrRP/PrRPR system may participate in the neuroendocrine control of fish reproduction. These results, taken together, suggest that PrRPs might act as a coupling factor in feeding metabolism and reproductive activities in teleost.

## Introduction

Prolactin-releasing peptide (PrRP), as a vital anorexigenic peptide, played an important role in food intake and energy metabolism (1–3). In mammals, central administration of PrRP could reduce food intake and body weight (2). In addition, PrRP-receptor-knockout (GPR10^-/-^) mice tended to become obese and showed conspicuous increases in body weight (4), in which attached fat mass increased and energy expenditures decreased (5). Similarly, the PrRP-knockout rodents could also display adiposity and hyperphagia (1). The potential mechanisms of the anorexigenic effect of PrRP were considered to be a pivotal intermediary in the satiety of signals produced by cholecystokinin (CCK), which could activate PrRP neurons in the hypothalamus (6–8). In addition, recent studies have also found that the anorexigenic action of PrRP was mainly relied on the leptin signal (9). Similar to a mammal, in goldfish, IP or intra-cerebroventricular (ICV) administration of PrRP could dose-dependently inhibit food intake, meanwhile feeding could significantly induce hypothalamus PrRP mRNA expression (10). However, little is known about the regulatory mechanism of feeding-induced hypothalamus PrRP expression.

PrRP was originally isolated from the hypothalamus and identified as a stimulator of prolactin (PRL) release in rats (11). Recent studies further found that PrRP is implicated in many physiological actions, including cardiovascular regulation (12, 13), stress response (14–16), sleep regulation (17, 18), growth performance (19), and reproduction in mammals (20). In male rats, ICV injection of PrRP could significantly stimulate LH release in plasma (20). However, the reproductive functions of PrRP in teleost have not been identified.

Generally, reproduction needs adequate energy to activate the hypothalamus-pituitary-gonadal (HPG) axis (21), namely higher food supply tends to promote reproduction, while the lower delays (22, 23). The connection between energy balance and reproduction had been demonstrated in detail mammals (21) and teleost (24). Recent studies have certified that hypothalamic neuropeptides, which are implicated in energy metabolism and food intake and involved in fertility, including gonadotropin-releasing hormone (GnRH) (25, 26), kisspeptin (27, 28), and gonadotropin-inhibitory hormone (GnIH) (29–31), served as a crucial mediator between metabolism and reproduction.

In the present study, we initially supposed that PrRP should be a potential mediator between reproduction and metabolism. To confirm this hypothesis, grass carp were used as the model to examine the functional role of PrRP in metabolism and reproduction. Firstly, we found that feeding could significantly induce hypothalamus PrRP mRNA expression, which indicated that PrRP should be an anorexigenic peptide in teleost. We further confirmed the functional role of PrRP in the regulation of pituitary gonadotropin hormone synthesis and secretion. These results will provide a novel idea that PrRP should also be a mediator between feeding metabolism and reproduction.

## Materials and Methods

### Animals and Chemicals

The tested grass carp (*Ctenopharyngodon idellus*) were purchased from a local market and kept in a 40-liter holding pond under a 12-hour light/12-hour dark photoperiod at optimum temperature. To consider the inconspicuous sexual characteristics of prepuberal grass carp, the temporary reared fish which mixed sexes, were employed for pituitary cell preparation and vivo test. All experimental procedures were approved by the Huazhong Agricultural University Administrative Panel for Laboratory Animal Care (Ethical Approval No. HBAC20091138; Date: 15 November 2009). Grass carp PrPR1 (SPEIDPFWYVGRGVRPIGRF-NH_2_), PrPR2 (DPNIDAVWYKGRG IRPVGRF-NH_2_), and human PrRP (TPDINPAWYASRGIRPVGRF-NH_2_, namely hPrRP) were synthesized by GeneScript (Piscataway, NJ). Meanwhile, the full-length open reading frame (ORF) of human PrRP receptor (hPrRPR) was obtained from the NCBI database (NO: NM_004248.3), as well as cloned into pcDNA3.1(-) Vector (Invitrogen). The signal inhibitors, namely MDL12330A, H89, U73122, GF109203X, 2-APB, KN62, nifedipine, calmidazolium (CMZ) were obtained from Calbiochem (San Diego, CA) (the background information for medicine used in *in vitro* experiments see **Supplementary Table S1**). The seventeen β-estradiol (E2, CAS NO: 50-28-2, Sigma-Aldrich) were obtained and diluted using ethanol. The rabbit PrRP primary antibody was acquired by injecting synthesized PrRP and purified antiserum from Dia-An Biotech, Inc (Wuhan, China). The synthetic peptides of PrRP1, PrRP2, and hPrRP were dissolved in ultrapure water at a final concentration of 1mM, while the rest of the medicine were subpackaged at 10mM concentration using dimethylsulfoxide (DMSO) and stored at a temperature lower than -80°C. Before drug treatment, they were diluted to working concentrations in advance by culture medium *in vitro* test, whereas in normal saline for fish *in vivo* test.

### Molecular Cloning and Tissue Expression of Grass Carp PrRPs and PrRP-Rs

To clone the two ligands and four receptors of PrRP in grass carp, total RNA was extracted from grass carp hypothalamus and pituitary, and reverse transcribed with HifairTM III 1^st^ Strand cDNA Synthesis Kit (Yeasen, Shanghai, China). Meanwhile, we used specific primers designed for the putative sequences of PrRPs ligands and receptors to clone the whole target gene of PrRPs and PrRP-Rs, respectively (**Supplementary Table S2**). Sequence alignment and phylogenetic analysis of grass carp PrRPs and PrRP-R were conducted with ClustalX 2.1 and MEGA 7.0. The 3-D structure for grass carp PrRPs and PrRP-Rs were predicted and modeled by using the I-TASSER and SWISS-MODEL, respectively. To illuminate the physiological functions of PrRPs and PrRPRs system in grass carp, the total RNA of the brain was divided into several parts, namely olfactory bulb, telencephalon, optic tectum, cerebellum, medulla oblongata, hypothalamus, and pituitary, as well as vital peripheral tissues. These were extracted and the cDNA samples were obtained by reverse transcription, then specific primers were used for these target genes to test the expression level by real-time PCR (RT-PCR) and gel electrophoresis (**Supplementary Table S3**). In these studies, β-actin was performed as an internal reference.

### Immunofluorescence Staining of PrRP1 in Grass Carp Hypothalamus

To verify PrRP cellular expressing distribution and functional region in grass carp, the hypothalamus of grass carp was isolated, obtained, and fixed in 4% paraformaldehyde. After fixation, the hypothalamus was dehydrated by gradient ethanol, then hyalinized in xylene and embedded with paraffin wax. The hypothalamus was sectioned under the condition of maximum longitudinal section and a thickness of 4 μm by a slicer (LEiCA, Shanghai, China). In the immunofluorescence experiment, after dragging wax and rehydration, the tissue section was soaked in citric acid antigenic repair buffer and microwaved from high to medium heat to accomplish antigen retrieval. To block nonspecific sites, the sections were incubated with 5% normal goat serum (Yeasen) for 1h at ambient temperatures. The sections were incubated with PrRP1 antibody (1:800) overnight at 4°C. Then after washing sections five times with 1×PBS the second day, added Alexa Fluor 594 Goat Anti-Rabbit IgG (1:200) (Yeasen). Finally, the section was subsequently counterstained with DAPI and sealed with an anti-fluorescence quenching mounting medium. The stained sections were imaged with a Leica SP8 confocal microscope.

### Functional Expression of Grass Carp PrRP-Rs and hPrRP-R in HEK-293T Cells

The ORFs of grass carp PrRP-Rs and human PrRP-R were subcloned into the pcDNA3.1(-) Vector (Invitrogen). Each receptor was transiently expressed in HEK-293T cells and subjected to treatment with PrRP1, PrRP2, and hPrRP. It was reported that PrRP could observably stimulate calcium mobilization and release in cells (32), therefore receptor activation was monitored by a pGL3-nuclear factor of activated T cells (NFAT)-RE-luciferase reporter system, which was established in a previous study (33). In brief, HEK-293T cells were cultured by Dulbecco’s Modified Eagle Medium (DMEM) supplemented with 10% fetal bovine serum (FBS, Gibico) in a 90-cm culture dish and incubated at 37°C with 5% CO_2_. After 2 days, these cells were digested and seeded in a 24-well cell culture plate at 3 ×10^5^ cells per well for 1 day before transfection. The cells were transfected by Lipofectamine in Opti-MEM with pGL3-NFAT-RE-luciferase reporters, in which green fluorescent protein (GFP) was used as an internal control. After transfection, PrRP-containing medium (PrRP-free medium was used as a control group) was added and the cells were incubated for an additional 24 h at 37°C. After washing using 1×PBS, the cells were dissolved by adding cell lysis buffer (Yeasen), and the cellular lysates were used to detect firefly luciferase and GFP luciferase activities by luciferase assay reagent (Promega) using multifunctional enzyme marking instrument (SpectraMax i3x).

### RNA-Seq and Bioinformatics

To investigate the direct effects of PrRP1 in grass carp pituitary, the pituitary cells were performed with the trypsin/DNase II digestion method which was described in previous studies (34). The dispersed cells were cultured into a 24-well cell culture plate at 2.5 million/0.8ml per well under 28°C, 5% CO2 with 5% FBS. After overnight incubation, PrRP1 (1 μM) was used to incubate pituitary cells for an extra 24 h under the same conditions. The total RNA was isolated subsequently by Trizol reagent (Yeasen), and DNase I was used to eliminate the interference of genomic DNA. After purity and concentration detecting by Nanodrop 2000 spectrophotometer, the high-quality RNA (RIN>8.0) was performed as a control group (three replicates) and the PrRP1-treatment group (three replicates) were sent to Majorbio Genome Center (Shanghai, China) for subsequent processing and sequencing on an Illumina HiSeq4000.

High quality clean reads were mapped to the grass carp genome using TopHat v2.0. Gene expression levels were assessed by the number of fragments per kilobase transcript per million fragments (FPKM). The *P* values were adjusted using Benjamini and Hochberg’s approach for controlling the false discovery rate (FDR<0.05). Different gene expressions (DEGs) were found with the fold change (FC)>1.5 and an adjusted *P* value<0.05 as a standard of differentially expressed. Gene Ontology (GO) enrichment analysis of the DEGs was implemented by the GOseq R packages based on Wallenius non-central hyper-geometric distribution for adjusting gene length bias in DEGs (35). The Kyoto Encyclopedia of Genes and Genomes (KEGG) was shown using Goatools software (36).

### FIA and Quantitative Real-Time PCR for LH Secretion and mRNA Expression in Pituitary Cells

To detect the direct effects of PrRP on LH secretion and related hormone expression levels in the pituitary. After cell culture and drug-treatment, the culture medium was obtained to monitor LH release levels using a fluorescence immunoassay (FIA) system (37). For grass carp LH, biotinylated grass carp LH was used as the tracer for LH assay. The Costar 96-well black plate (Thermo Fisher Scientific, USA) was preprocessed with protein A and reading buffer which contained biotinylated grass carp LH and LH antibody. The protein sample was then added into wells respectively. After incubating overnight at 4°C, these wells were washed three times and horseradish peroxidase (HRP)-conjugate streptavidin was added subsequently for additional 1 h incubation. Then the solution was removed into individual wells and QuantaBlu Fluorogenic Peroxidase Substrate (Thermo Fisher Scientific) was added, which was used for fluorescence signal detection.

In parallel experiments, total RNA was extracted using Trizol, then reversely transcribed, and subjected to quantitative real-time PCR (Q-RT-PCR) for LHβ, FSHβ, GtHα, DRD2 mRNA measurement using an ABI 7500 Q-RT-PCR system (Biosystems, USA). (See the **Supplementary Table S3** for primer sequences and PCR conditions for the respective gene targets). In these experiments, serial gradient dilution of plasmid DNA of grass carp LHβ were used as a standard for data calibration, and β-actin was used as an internal control.

### PrRPs Induced LHβ Promoter Activity in HEK-293T

To verify whether the direct regulation of PrRPs by activating their specific G-protein-coupled receptors (GPCRs) to cause LHβ mRNA expression and protein release, the sequence of LHβ promoter was obtained from NCBI (Gene ID: EF194763) and its putative transcription factor binding regions were analyzed with the online services JASPAR database and Signal Scan. The LHβ promoter (1141bp) was isolated by specific primers from grass carp genomic DNA (**Supplementary Table S2**). After purification, the PCR products were firstly cloned into T-easy vector (Promega), followed by double restriction enzymes digestion and sub-cloned into pGL3 Basic vector (Promega) used for luciferase assay. Besides, the PrRP-Rs were cloned into the pcDNA3.1(-) vector. HEK-293T cells were transiently co-transfected with the luciferase-express reporter constructs carrying the 5’ promoter of grass carp LHβ gene and PrRP receptor plasmids as well, meanwhile, a GFP luciferase-expression vector was used as an internal control.

### *In Vivo* Effect of PrRP Treatment

After temporarily keeping grass carp in a well-aerated 30-liter storage pond with the one-meal-per-day feeding schedule for 14 days to domesticate, a single-dose IP injection of PrRPs was operated as described previously (38). After that, a time-course experiment of injecting PrRPs was performed to detect serum LH levels. In brief, the tested grass carp were fast anesthetized by MS222 (Sigma) and weighed the body weight respectively. Then, injected solution (normal fish saline (0.7% NaCl) was used as a control, and the injection dose of PrRPs is in accordance with 100 ng/g body weight) was injected into the peritoneal cavity softly. Fish were returned to the storage pond and recovered from anesthesia. In this process, the pituitaries were collected at 24 h, and blood was taken at 3 h, 6 h, and 12 h, respectively. The total RNA was extracted from the pituitary by Trizol and reverse transcribed into cDNA to detect the mRNA levels of LHβ, FSHβ, GtHα. Meanwhile, after centrifugation, serum was extracted and used to detect LH release.

### Postprandial Changes in PrRPs mRNA Expression and Blood Glucose Level

Grass carp entrained with a one-meal-per-day feeding schedule (with six fish per group) was provided with fish feed at 9:00 Am (taken as 0 h). On an experimental day, after food supply and being anesthetized by MS222, tail cutting was performed on the fish and we measured blood glucose levels at 0, 1, 3, and 6 h by blood glucose meter (ACCU-CHEK Performa, Roche), respectively. The brains were harvested and stored in liquid nitrogen, respectively. Then the total RNA was extracted and reverse transcribed to monitor the PrRPs mRNA level expression by RT-PCR system, besides, internal control was tested using β-actin in this test.

### Primary Brain Cells Culture

To verify the potential upstream regulatory element which led to the PrRPs-induced LH release and mRNA expression, the primary grass carp brain cells were prepared by a method described in previous studies (38). In brief, 13-14 grass carp brains were obtained and separated into the required brain areas including telencephalon, hypothalamus, and optic tectum, which were cut into 0.8 mm tissue fragments using Mcilwain tissue chopper (Cavey laboratory). After washing with Minimum Essential Medium (MEM, Gibco) three times, the brain fragments were incubated with 6 mg/ml trypsin (Sigma) dissolved in the medium under the conditions of 28°C, 48 rpm for 30 min. This process ended by transferring the brain fragment into 2.5 mg/ml trypsin inhibitor (Sigma) for 5 min and sequentially incubated in Ca^2+^-free medium (Gibco) supplemented with 0.05 M ethylene diamine tetraacetic acid (EDTA, Sigma) and 0.1 mg/ml DNaes II (Sigma). The brain fragments were filtered using a 30 μm filter screen and cultured into a 24-well cell culture plate under 28°C, 5% CO_2_ concentration. After culturing for 3 days, the medium was replaced with NeuroGro medium (BasalMedia) which contained 1:50 diluted 50X B-27 serum free supplement (Gibco), 1:40 diluted GlutaMAX (Gibco), 200 U/ml penicillin, and 0.2 mg/ml streptomycin (Yeasen). After another 2 days of incubation, a single-dose E2 treatment (1 μM) was performed on the cells. Then total RNA was extracted by Trizol reagent and reversely transcribed, to examine PrRPs and PrRP-Rs mRNA expression level. The GnRH2 and GnRH3 mRNA were detected as a positive control.

### Data Transformation and Statistical Analysis

In this study, the condition of quantitative real-time PCR of LHβ, FSHβ, GtHα, DRD2, GnRH2, and GnRH3 mRNA, was that the data calibration restricted curves with a dynamic range of 10^5^ and correlation coefficient >0.95 with ABI7500 software. The β-actin mRNA was used as an internal parameter to eliminate random error. Therefore, genes mRNA data as well as LH release data were easily converted as a percentage of the mean value (as “% Ctrl”). The data presented (as Mean ± SEM) collected results from 4-8 parallel group experiments and analyzed with ANOVA. The differences between each group were significant at P-value<0.05 by labeling diverse letters.

## Results

### Molecular Cloning and Sequence Analysis of PrRPs and PrRP Receptors in Grass Carp

**Figure 1:**
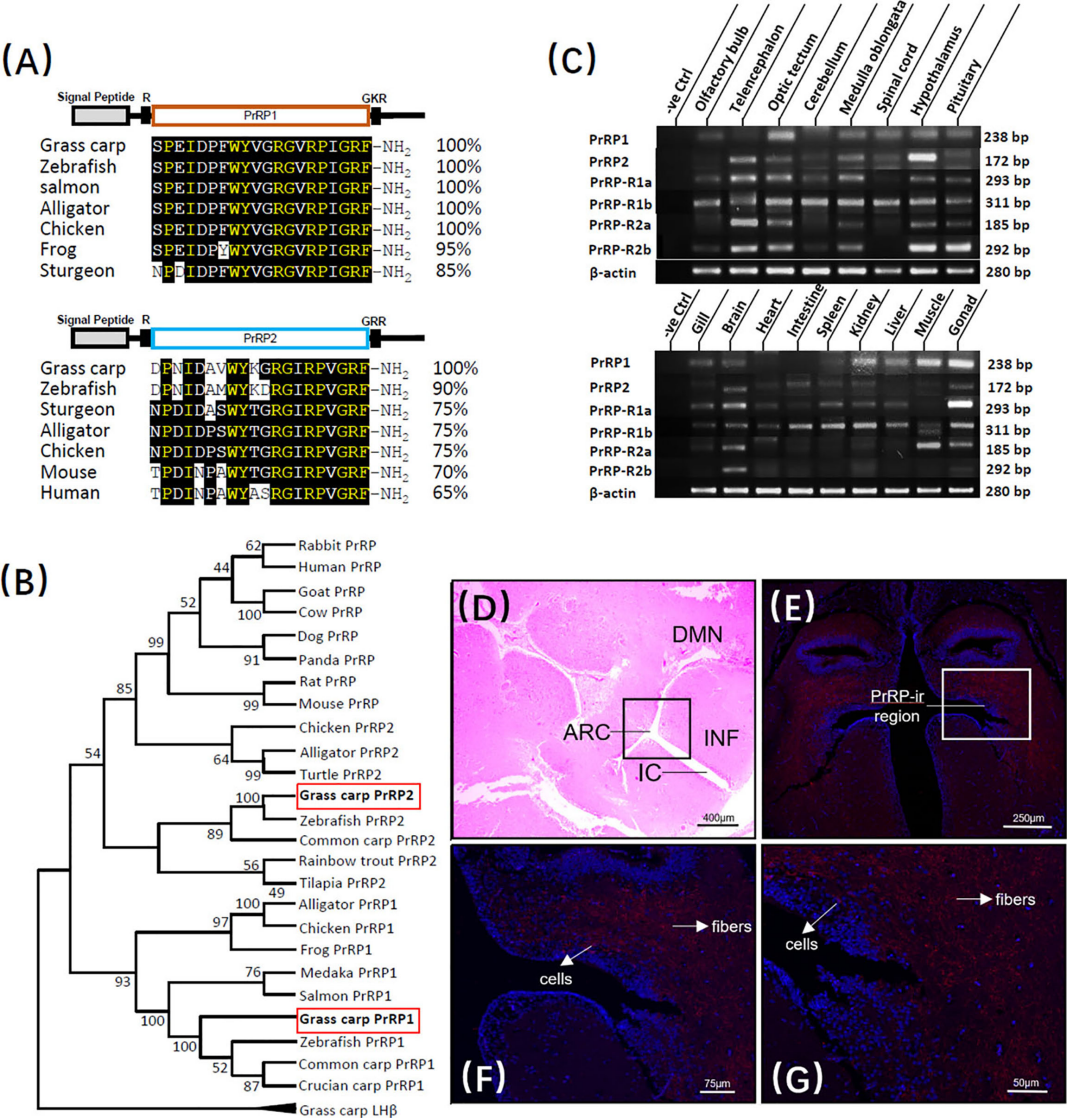
Sequence analysis and tissue distribution of grass carp PrRPs. **(A)** The mature peptides sequence alignment of PrRPs with counterparts in other species. The conserved amino acid sequences are processed into a black background, whereas the same amino acid residues compared with PrRP1 and PrRP2 are marked in yellow. **(B)** Phylogenetic analysis of PrRPs from mammal or non-mammal vertebrates are generated with neighbor-joining (MEGA 6.0) and grass carp PrRPs are highlighted in the red frame. **(C)** Tissue distribution of PrRP1, PrRP2, PrRP-R1a, PrRP-R1b, PrRP-R2a, PrRP-R2b in grass carp peripheral tissues (on the bottom) and various brain subregions (on the top). Total RNA was extracted, reverse transcription, and performed to RT-PCR using specific primers, the results have been intercepted and spliced according to corresponding PCR product size. Besides, the transcript level of β-actin was considered as an internal control. **(D)** HE Staining was performed on a horizontal section of the hypothalamus. **(E–G)** PrRP-ir cells (red) were mainly observed in the ARC region and PrRP-ir fibers were in ARC, DMN region by immunofluorescence staining. In this study, DAPI was used to marked the cells (blue) in grass carp hypothalamus. Abbreviations: ARC, arcuate nucleus; DMN, dorsomedial nucleus; INF, infundibulum; IC, infundibulum cavity. Scale bars: 400 μm in **(D)**, 250 μm in **(E)**, 75 μm in **(F)** and 50 μm in **(G)**.

The full-length of grass carp PrRP1 (GenBank NO.: MK078510) and PrRP2 (GenBank NO.: MK078511) were cloned using specific primers, and sequence analysis indicated that the ORF of PrRP1, PrRP2 possessed 354bp and 297bp in size as well as encoding 118- and 99-amino acid protein precursor, respectively (**Supplementary Figure S1**). Similar to non-mammal vertebrates, grass carp contain two separate PrRP subtypes, severally encoding one mature peptide. PrRP1 contained the 20-aa sequence (SPEIDPFWYVGRGVRPIGRF-NH_2_), meanwhile PrRP2 contains 20-aa peptide (DPNIDAVWYKGRGIRPVGRF-NH_2_) as well, which were both with the common signature motif (RF-NH_2_) in the C terminus (**Supplementary Figure S1**). At the protein level, we contrasted the mature peptides of PrRP in each species, the results showed that as the peptide specific merely existed for non-mammalian vertebrate, grass carp PrRP1 hormone mature peptide displayed 100% identical to the counterparts of zebrafish, salmon, alligator, chicken when showed 95% and 85% identical to frog and sturgeon, respectively. Meanwhile, as the mammalian PrRP homologue, the PrRP2 mature peptide of grass carp revealed 65% identity contrasted with the human counterpart and 70% for mice. Besides, grass carp PrRP2 share a higher similarity among nonmammalian vertebrates: 90%, 75%, 75%, and 75% similarity when compared to the corresponding peptides for zebrafish, sturgeon, alligator, and chicken (**Figure 1A**). We generated a phylogenetic tree containing the identified grass carp PrRPs precursors and other PrRPs sequences which had been cloned or predicted previously from mammal or non-mammal vertebrates. Phylogenetic analysis illustrated that the two different subtypes of PrRPs were clustered into two separate branches, in which PrRP1 and PrRP2 both had high similarity with non-mammalian vertebrates while PrRP2 was closely related to mammalians but PrRP1 was unique for nonmammalian vertebrates (**Figure 1B**).

In addition to PrRPs, grass carp PrRP-R1a (352aa, GenBank NO.: MK078513), PrRP-R1b (358aa, GenBank NO.: MK078514), PrRP-R2a (371aa, GenBank NO.: MK078512), and PrRP-R2b (354aa) were also isolated from grass carp brain and pituitary, respectively (**Supplementary Figures S2, S4, S6, S8**). As the members of the GPCR group, the amino acid sequence of the four newly cloned PrRP receptors could be structured into seven transmembrane domains (TMD 1 to 7) with three intracellular loops and three extracellular loops, together with an endocellular C-terminal and extracellular N-terminal tail (**Supplementary Figures S3, S5, S7, S9**). Based on amino acid sequences, phylogenetic analysis using the neighbor-joining method revealed that the four PrRP receptors were orthologs in the carp model and were clustered in separated clades, of which, the PrRP-R2a and PrRP-R2b could be clustered in the same brand with mammalian PrRP-R, whereas PrRP-R1a and PrRP-R1b were clustered into two distinct brands with other nonmammalian species (**Supplementary Figure S10**).

### Tissue Distribution of PrRPs and Its Four Receptors in Grass Carp

To demonstrate the potential physiological roles of the PrRPs/PrRP-Rs system, the transcript level of PrRPs and their receptors were all examined by using RT-PCR in vital peripheral tissues and brain areas of grass carp. At the peripheral tissues level, transcript signals for PrRP1 were mainly detected in the gonad, muscle, kidney, liver, gill, and brain, while PrRP2 was highly detected in the brain, intestine, kidney, and gonad. In the brain, high transcript levels of PrRP1 were detected in the optic tectum, together with a medium expression in the medulla oblongata, spinal cord, hypothalamus, and pituitary. However, PrRP2 were abundantly expressed in the telencephalon, hypothalamus, medulla oblongata, and optic tectum, with a modest level in the olfactory bulb, cerebellum, spinal cord, and pituitary (**Figure 1C**).

Interestingly, RT-PCR signals for PrRP-R2a and PrRP-R1a were specifically located in the brain and gonad. However, PrRP-R1b was widely distributed in the selected tissues, and PrRP-R2b merely revealed a high level in the brain. Within the various brain subregions, the transcript signals of all four receptors were detected in the pituitary, and PrRP-R1a, PrRP-R2a, PrRP-R2b displayed high expressed levels in the telencephalon, optic tectum, medulla oblongata, hypothalamus, and pituitary, whereas PrRP-R1b were abundantly expressed in the selected brain areas (**Figure 1C**).

### Immunofluorescence Staining of PrRP1 in Grass Carp Hypothalamus

Grass carp hypothalamus section was disposed to hematoxylin-eosin (HE) staining in a previous study (39), in which a randomly distributed enormous number of secretory cells were surrounded by bits of nerve fiber, neuron, and capillary (**Figure 1D**). Based on this, we localized the PrRP1 expressing cellular sites in the grass carp hypothalamus by Immunofluorescence (IF) reaction with a high specific antibody. The result revealed that the PrRP-immunoreactive (PrRP-ir) region (in red) is principally located in the arcuate nucleus (ARC) and dorsomedial nucleus (DMN) region (**Figure 1E**). Scattered PrRP-ir cells projected chiefly in the ARC region (**Figures 1F, G**), and abundant PrRP-ir fibers were mainly observed in a region which extended from ARC to DMN (**Figures 1F, G**). Finally, all the cells were indicated to blue by using DAPI as a reference.

### Functional Analysis of Grass Carp PrRP-rs and Human PrRP-r in HEK-293 Cells

**Figure 2:**
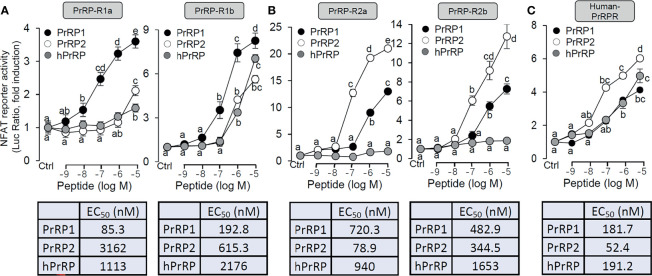
Functional expression of grass carp PrRP-Rs and human PrRP-R in HEK-293T cells. To indicate the ligand-receptor selectivity of the latterly cloned grass carp PrRP1, PrRP2, and four receptors, together with synthetic hPrRP and hPrRPR, a NFAT-luc reporter system was used in HEK-293T cells which were treated with various concentrations of PrRPs for 24 h, to detect the luciferase activity of PrRP-R1a, PrRP-R1b **(A)**, PrRP-R2a, PrRP-R2b **(B)** and hPrRPR **(C)**. The EC_50_ values of PrRPs for each receptor were calculated by GraphPad Prism 7. Data presented are expressed as mean ± SEM, and the differences between groups were significant at P-value<0.05 by labeling diverse letters.

Functional expression analysis of PrRP-rs was used to indicate the ligand-receptor selectivity of the latterly cloned PrRPs and PrRP-rs in HEK-293 cells. Previous studies report that activation of mammalian PrRPr could induce intracellular calcium mobilization (11, 40). Therefore, a pGL3-NFAT-RE-luciferase reporter system, which could monitor the changes in intracellular calcium concentration and could stably express PrRP-Rs, was used in the present study. The result showed that both grass carp PrRP1 and PrRP2 could dose-dependently induce NFAT-luc activity *via* activation of all grass carp PrRP-Rs (**Figures 2A, B**). Interestingly, for PrRP-R1a and PrRP-R1b, grass carp PrRP1 (with EC_50_ at 85.3 nM and 192 nM, respectively) was found to be notably more potent in triggering stimulatory effect compared to PrRP2 (with EC_50_ at 3162 nM and 615.3 nM, respectively) (**Figure 2A**). Meanwhile, PrRP-R2a and PrRP-R2b were more sensitive to the activation by PrRP2 (with EC_50_ at 78.9 nM and 344.5 nM, respectively) compared to PrRP1 (with EC_50_ at 720.3 nM and 482.9 nM, respectively) (**Figure 2B**). However, hPrRP could merely activate grass carp PrRP-R1b when low activation capacity was shown to other grass carp PrRP-Rs by hPrRP (**Figures 2A, B**). Besides, hPrRPR was activated by both grass carp PrRP1 (with EC_50_ at 181.7 nM), PrRP2 (with EC_50_ at 52.4 nM), and hPrRP (with EC_50_ at 191.2 nM), indeed grass carp PrRP ligand even indicated a more robust activation capability than hPrRP in high concentration (**Figure 2C**).

### Transcriptomic Analysis of PrRP1 in the Pituitary

**Figure 3:**
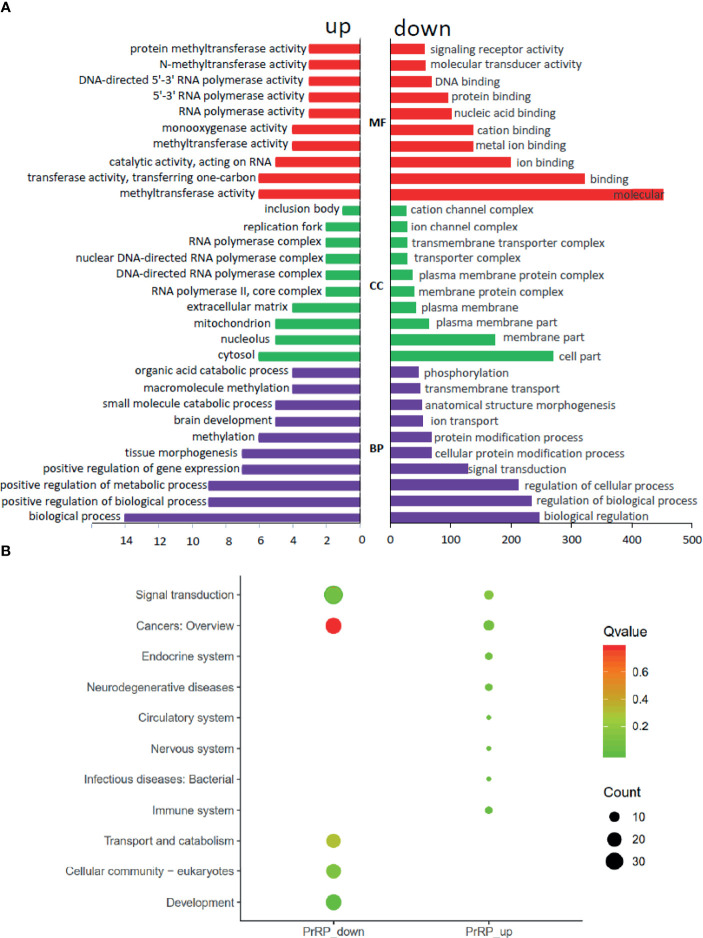
Gene ontology (GO) analysis and Kyoto Encyclopedia of Genes and Genomes (KEGG) analysis based on the transcriptome. **(A)** GO analysis revealed that the selected DEGs, which satisfied the conditions of FPKM > 20, FDR < 0.05, FC > 1.5, or FC < 0.8 were made up of three parts, namely cellular component (CC), biological process (BP), and molecular function (MF). **(B)** 285 DEGs were classified into the top 10 enriched pathways using KEGG analysis which included up-regulation and down-regulation DEGs. Q value, Recalibration of P value. Count, the number of DEGs.

To investigate the direct pituitary action of PrRP1, primary cultured pituitary cells derived from prepuberty grass carp were treated with 1 μM PrRP1. After 24-hour treatment, high-throughput RNA-seq was utilized to compare the mRNA expression difference between the control and PrRP1 treated groups. According to the fragments per kilobase of exon per million mapped (FRKM) reads method, the differential expression genes (DEGs) were identified, and the gene abundances were quantified. 291 DEGs were filtrated under the condition of FPKM>20, FDR<0.05, which includes 241 up-regulated (FC>1.5) genes and 50 down-regulated (FC<0.8) genes. GO analysis showed that these DEGs were made up of three parts: cellular component, biological process, and molecular function (**Figure 3A**). In the molecular function category, ‘molecular’, ‘binding’, ‘ion binding’, ‘metal ion binding’, ‘cation binding’ were the amplest. In another aspect, ‘cell part’, ‘membrane part’, ‘plasma membrane part’, ‘plasma membrane’, ‘membrane protein complex’ were the most abundant GO clause in the cellular component (**Figure 3A**). In addition, the GO enrichment analysis of the biological process was divided into two main contents composed of the top 37 up-regulated DEGs (**Table 1**) and top 36 down-regulated DEGs (**Table 2**), which elaborate the genes, FC, FDR, description and biological process of each DEGs, respectively.

**Table 1:** The up-regulated genes induced by PrRP1 in grass carp pituitary cells.

Gene	Description	FC	FDR	Go-biological process
PSMD4	proteasome26S subunit 4b	1.32	2.93E-04	ubiquitin-dependent process
NDUFC2	NADH dehydrogenase 1 subunit c2	1.28	3.26E-05	ubiquinone biosynthetic process
GtHa	glycoprotein hormones, alpha polypeptide	1.28	1.30E-09	signal transduction
PRDX5	peroxiredoxin-5	1.22	6.94E-04	single-multicellular organism process
LHβ	luteinizing hormone beta subunit	2.90	7.03E-114	signal transduction;
FSHβ	follicle stimulating hormone beta subunit	1.28	6.98E-07	signal transduction
HSD3β	3beta-hydroxysteroid dehydrogenase	2.19	1.26E-02	steroid biosynthetic process
RPL36	PREDICTED: 60S ribosomal protein L36	1.54	2.01E-20	ribosome biogenesis
RPL22	60S ribosomal protein L22-like	1.27	3.79E-04	ribosome biogenesis
SNU13	NHP2 non-histone chromosome protein	1.22	2.79E-03	ribosome biogenesis
RBM8A	RNA-binding protein 8A	1.24	3.73E-04	regulation of translation
EDNRB	endothelin B receptor	1.22	5.48E-04	regulation of blood pressure
NDUFB8	NADH dehydrogenase	1.22	5.90E-04	proton transport
SHFM1	26S proteasome complex subunit DSS1	1.31	4.18E-06	proteolysis
UFC1	ubiquitin-fold modifier-conjugating 1-like	1.25	1.50E-04	protein ufmylation
OST2	protein glycosyltransferase subunit DAD1	1.35	7.48E-07	protein N-linked glycosylation
HSPE1	10 kDa heat shock protein	1.43	1.76E-08	protein folding
GRPE	grpE protein homolog 2	1.23	5.93E-04	protein folding
CCT4	T-complex protein 1 subunit delta-like	1.35	1.54E-06	protein folding
PFDN4	prefoldin sub`unit 4 isoformX1	1.21	3.05E-03	protein folding
NOP10	H/ACA ribonucleoprotein complex subunit 3	1.41	9.41E-06	primitive hemopoiesis
CD9	CD9 molecule b	1.32	8.96E-07	posterior lateral line development
TCEB1	Crystal Structure of Socs-2 With Elongin-B	1.20	1.79E-03	positive regulation of transcription
ATP6S14	V-type proton ATPase subunit F	1.23	4.53E-04	oxidative phosphorylation
QDPR	quinoid dihydropteridine reductase	1.31	7.35E-06	oxidation-reduction process
H3	putative H3 histone family 3B variant 1	1.21	9.95E-04	nucleus organization
SRP9	signal recognition particle 9	1.46	2.38E-08	regulation of translational elongation
SERPINB	Leukocyte elastase inhibitor	1.28	2.90E-05	regulation of endopeptidase activity
SF3B14	splicing factor 3B subunit 6	1.30	6.22E-06	mRNA splicing, *via* spliceosome
ATOX1	ATX1 antioxidant protein 1 homolog	1.21	9.69E-04	metal ion transport
COX7C	cytochrome c oxidase subunit 7C	1.35	2.19E-06	hydrogen ion transport
COX7A	cytochrome c oxidase, subunit VIIa 2a	1.24	2.18E-03	hydrogen ion transport
SNRPD1	small nuclear ribonucleoprotein Sm D1	1.26	5.49E-04	heart development
NDUFB3	NADH dehydrogenase	1.47	1.18E-07	electron transport chain
RBX1	RING-box protein 1	1.20	4.32E-03	biosynthetic process
ATPeF0F6	ATP synthase-coupling factor 6	1.44	8.10E-10	ATP synthesis coupled transport
QCR7	cytochrome b-c1 complex subunit 7-like	1.35	3.60E-07	angiogenesis

FC, fold change; FDR, false discovery rate.

**Table 2:** The down-regulated genes induced by PrRP1 in grass carp pituitary cells.

Gene	Description	FC	FDR	Go-biological process
EYA1	eyes absent homolog 1 isoform X3	0.78	9.81E-04	tyrosine metabolic process
SYT1	synaptotagmin Ia isoform X1	0.74	9.57E-05	transport
SSTR3	somatostatin receptor type 3	0.77	1.53E-03	synaptic transmission
SPOCK	testican-1-like	0.75	1.88E-03	signal transduction;
PER	period circadian protein homolog 3	0.75	1.10E-05	signal transduction
CYFIP	cytoplasmic FMR1-interacting protein 2	0.79	9.11E-04	retina layer formation
HSPA1_8	heat shock 70 kDa protein	0.77	2.73E-04	response to stress
FOXO1	forkhead box protein O1-A	0.80	3.57E-03	regulation of transcription
RGL2	nucleotide dissociation stimulator-like 2	0.79	2.78E-03	regulation of GTPase activity
TRIP12	E3 ubiquitin-protein ligase TRIP12	0.76	1.31E-05	protein ubiquitination
CAMK1	calmodulin-dependent protein kinase type 1D	0.72	8.84E-05	protein phosphorylation
SIK3	threonine-protein kinase SIK3 homolog	0.68	3.95E-06	protein phosphorylation
DRD2	dopamine D2 receptor	0.69	6.78E-05	synaptic transmission
PTPRN	protein tyrosine phosphatase Nb isoform X1	0.75	2.20E-04	protein dephosphorylation
EPRS	bifunctional aminoacyl-tRNA synthetase	0.80	4.33E-04	prolyl-tRNA aminoacylation
KCNT2	potassium channel subfamily T member 2	0.77	1.65E-04	potassium ion transport
GBF1	guanine nucleotide exchange factor 1	0.76	2.01E-05	positive regulation of GTPase activity
KDM5	lysine-specific demethylase 5C	0.79	6.59E-04	oxidation-reduction process
CPLX1_2	complexin-2	0.79	7.55E-03	neurotransmitter transport
PPP1R37	Si:ch211-234g24.1 protein	0.80	1.24E-03	regulation of phosphatase activity
BAI1	brain-specific angiogenesis inhibitor 1-like	0.75	4.51E-07	negative regulation of angiogenesis
PTBP3	polypyrimidine tract-binding protein 3	0.72	3.95E-05	mRNA processing
CHK	choline kinase alpha-like	0.79	2.67E-03	metabolic process
ZBTB38	zinc finger and BTB protein 38	0.68	1.52E-10	mediolateral intercalation
CAMK2	calmodulin-dependent protein kinase type II	0.77	5.43E-04	heart jogging
DNM	Dynamin-1	0.78	3.20E-04	GTP catabolic process
REM2	RAS (RAD and GEM)-like GTP binding 2	0.64	1.23E-09	GTP catabolic process
REM2	RAS (RAD and GEM)-like GTP binding 2	0.71	1.55E-04	GTP catabolic process
SDC3	syndecan-3	0.80	2.25E-02	epiboly involved in gastrulation
UPF1	regulator of nonsense transcripts 1	0.80	1.29E-03	embryo development
SREBP2	sterol regulatory element-binding protein 2	0.77	3.83E-05	cholesterol metabolic process
TBX19	T-box transcription factor TBX19-like	0.70	4.03E-05	cell fate commitment
DDR1	discoidin domain-containing receptor 1	0.78	2.88E-05	cell adhesion
SCARB2	lysosome membrane protein 2	0.78	9.75E-04	cell adhesion
PC	pyruvate carboxylase, mitochondrial-like	0.80	1.69E-02	aspartate metabolic process
SLC7A4	cationic amino acid transporter 4	0.74	2.44E-05	amino acid transmembrane transport

FC, fold change; FDR, false discovery rate.

To further understand the direct pituitary functions of PrRP1, annotated pathways of DEGs were analyzed using the Kyoto Encyclopedia of Genes and Genomes (KEGG) database. The results showed that 285 DEGs were enriched totally in the top 10 pathways, in which the up-regulated DEGs were mostly enriched in ‘Cancers: Overview’, ‘Signal transduction’, ‘Immune system’, together with the down-regulated DEGs were mainly enriched in ‘Signal transduction’, ‘Cancers: Overview’, ‘Signal transduction’ (**Figure 3B**).

### Regulation of Pituitary Hormones Secretion and mRNA Expression by PrRPs

**Figure 4:**
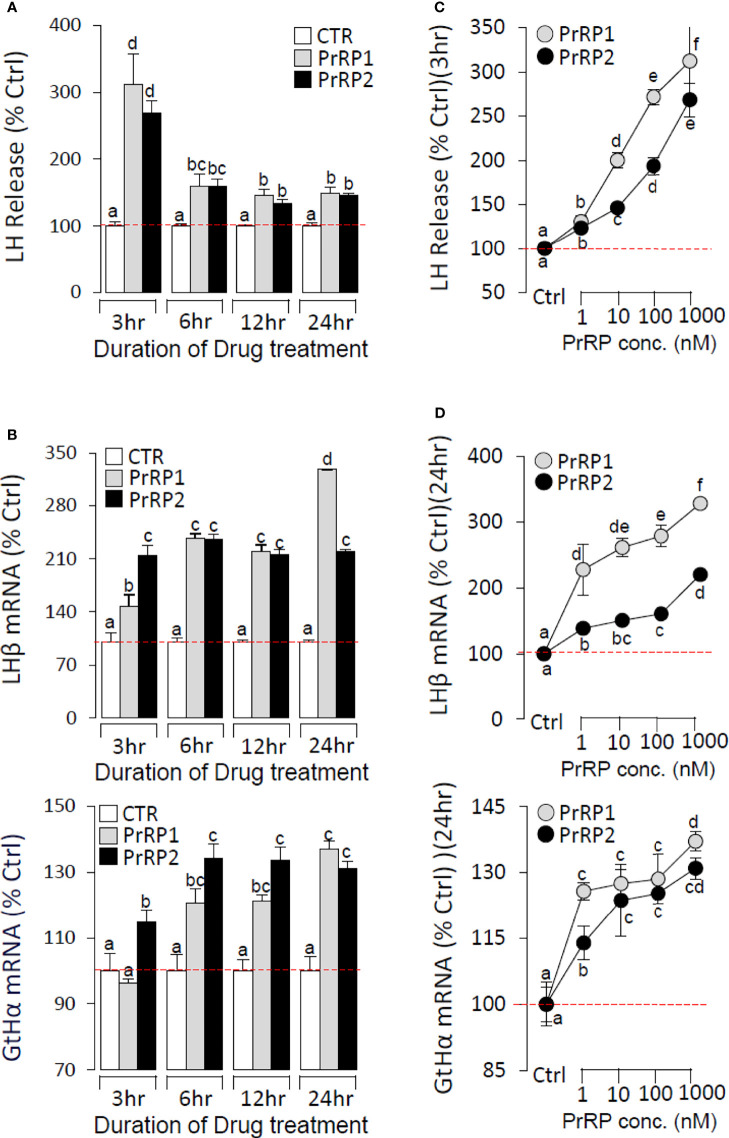
Novel pituitary actions of PrRPs on LH secretion and mRNA expression in grass carp pituitary cells. Time course of grass carp PrRP1 (1 μM) and PrRP2 (1 μM) treatment on LH release **(A)** and LHβ & GtHα mRNA expression **(B)** in grass carp pituitary cells. **(C)** Dose-dependence of 3-hr treatment with increasing levels of PrRP1 and PrRP2 (1-1000 nM) on LH release in grass carp pituitary cells. **(D)** Dose-dependence of 24-hr treatment with increasing levels of PrRP1 and PrRP2 (1-1000 nM) on LHβ & GtHα mRNA expression in grass carp pituitary cells. After drug treatment, the culture medium was collected for testing the LH secretion, and the remaining pituitary cells were extracted to total RNA, reversed transcription, and used for RT-PCR to detect the LHβ and GtHα mRNA expression. Data presented are expressed as mean ± SEM, and the differences between groups were significant at P-value < 0.05 by labeling diverse letters.

**Figure 5:**
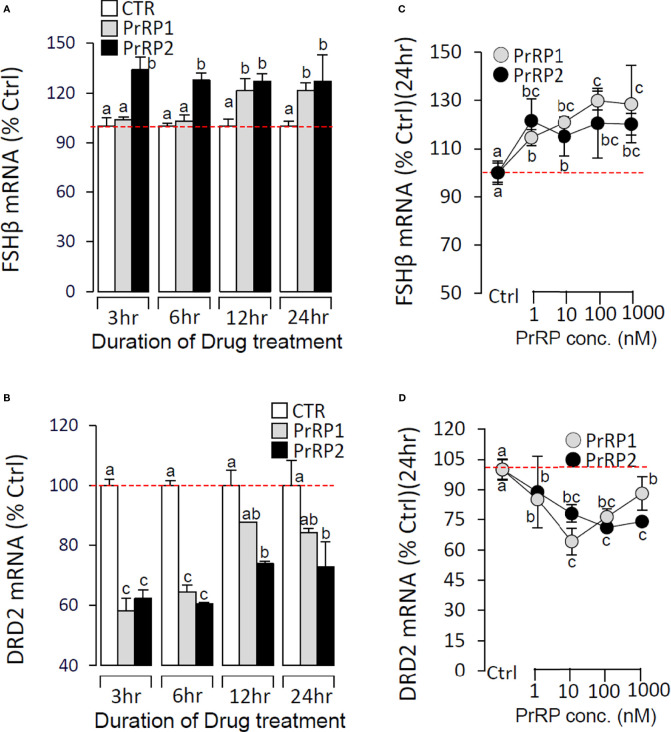
Effects of PrRPs on FSHβ and DRD2 mRNA expression in grass carp pituitary cells. Time course of grass carp PrRP1 (1 μM) and PrRP2 (1 μM) treatment on FSHβ **(A)** and DRD2 **(B)** mRNA expression in grass carp pituitary cells. Dose-dependence of 24-hr treatment with increasing levels of PrRP1 (1-1000 nM) and PrRP2 (1-1000 nM) on FSHβ **(C)** and DRD2 **(D)** mRNA expression in grass carp pituitary cells. After drug treatment, total RNA was isolated for real-time PCR of FSHβ and DRD2 transcript level using primers specific for the respective targets. Data presented are expressed as mean ± SEM, and the differences between groups were significant at *P*-value < 0.05 by labeling diverse letters.

To further confirm the direct pituitary actions of two PrRP subtypes, primary cultured pituitary cells were initially treated with PrRP1 (1 μM) for 24 h. The results indicated that PrRP1 could significantly induce LHβ, GtHα, and FSHβ mRNA expression but without effect on other pituitary hormones (PRL, SLα, SLβ, TSHβ, and GH) mRNA expression (**Supplementary Figure S11**). Furthermore, PrRP1 and PrRP2 (1 μM) could both significantly stimulate LH secretion in grass carp pituitary cells, which showed a transient peak of LH secretion at 3 h (**Figure 4A**). PrRPs could also significantly induce pituitary LHβ and GtHα mRNA expression from 3 to 12 h in a time-dependent manner (**Figure 4B**). In addition, PrRP1 and PrRP2 could trigger a mild up-regulation of FSHβ mRNA expression (**Figure 5A**). Interestingly, PrRPs could significantly inhibit pituitary DRD2 mRNA expression in a time-dependent manner (**Figure 5B**). In the dose-dependent test, a continuous gradient dilution of PrRP1 or PrRP2 (1-1000 nM) was incubated with grass carp pituitary cells for 24 h (LH release for 3 h), respectively. The results revealed that both PrRP1 and PrRP2 could induce LH secretion and mRNA expression in a dose-dependent manner (**Figures 4C, D**). Besides, the increasing dose (1 to 1000 nM) of PrRP1 and PrRP2 could also mildly stimulate FSHβ and significantly inhibit DRD2 mRNA expression in grass carp pituitary cells (**Figures 5C, D**).

### Signal Transduction for PrRPs-Induced LH Secretion and mRNA Expression in Grass Carp Pituitary Cells

**Figure 6:**
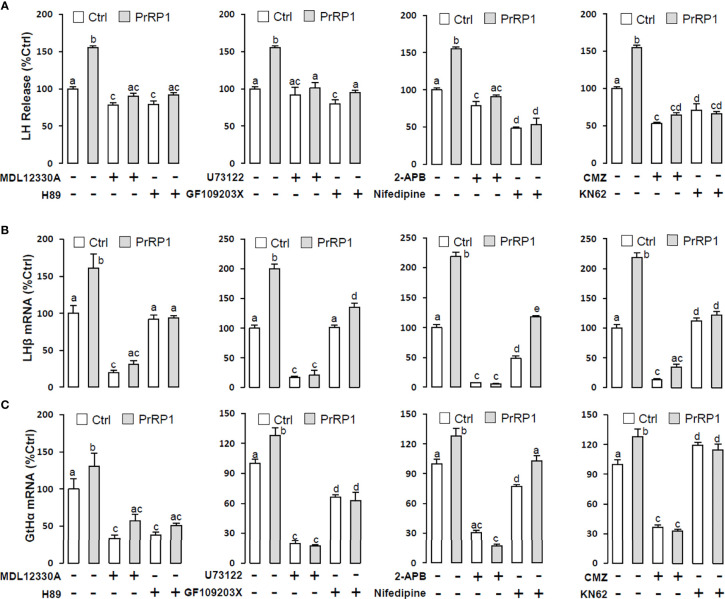
Signal transduction for PrRP1-induced LH secretion and mRNA expression in grass carp pituitary cells. Effects of co-treatment with AC inhibitor MDL12330A (20uM), PKA inhibitor H89 (20uM), PLC inhibitor U73122 (10 μM), PKC inhibitor GF109203X (20 μM), IP3 receptor blocker 2-APB (100 μM), VSCC inhibitor nifedipine (10 μM), CaM antagonist calmidazolium (1 μM) or CaMK-II blocker KN62 (5 μM) on PrRP1 (1 μM)-induced LH release **(A)**, LHβ **(B)** and GtHα **(C)** mRNA expression in grass carp pituitary cells for 24 h. After drug treatment, the medium was harvested for hormone release and total RNA was extracted from the remaining cells for real-time PCR of the respective genes. Data presented are expressed as mean ± SEM, and the differences between groups were significant at P-value < 0.05 by labeling diverse letters.

**Figure 7:**
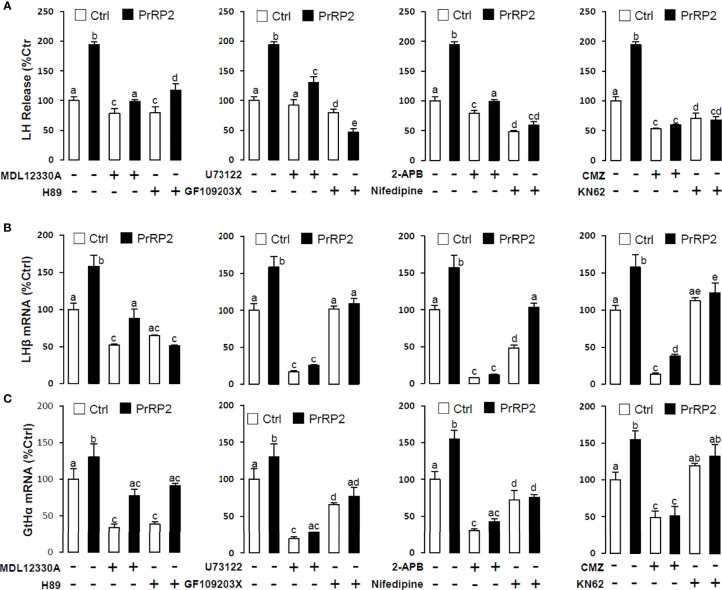
Signal transduction for PrRP2-induced LH secretion and mRNA expression in grass carp pituitary cells. Effects of co-treatment with AC inhibitor MDL12330A (20uM), PKA inhibitor H89 (20uM), PLC inhibitor U73122 (10 μM), PKC inhibitor GF109203X (20 μM), IP3 receptor blocker 2-APB (100 μM), VSCC inhibitor nifedipine (10 μM), CaM antagonist calmidazolium (1 μM) or CaMK-II blocker KN62 (5 μM) on PrRP2 (1 μM)-induced LH release **(A)**, LHβ **(B)** and GtHα **(C)** mRNA expression in grass carp pituitary cells for 24 h. After drug treatment, the medium was harvested for hormone release and total RNA was extracted from the remaining cells for real-time PCR of the respective genes. Data presented are expressed as mean ± SEM, and the differences between groups were significant at P-value < 0.05 by labeling diverse letters.

To reveal the signal transduction for PrRPs-induced LH secretion and mRNA expression, the inhibitors of AC/PKA and PLC/IP3/PKC signal pathway were used to examine the possible factors involved in cAMP-dependent cascades. The stimulatory effects of PrRPs on LH secretion and mRNA expression by PrRP1 (1 μM) or PrRP2 (1 μM) could be blocked by co-treatment with the AC inhibitor MDL12330A (20 μM) or PKA inhibitor H89 (20 μM) in grass carp pituitary cells (**Figures 6**, **7**). Similarly, the PLC inhibitor U73122 (10 μM), PKC inhibitor GF109203X (20 μM), and IP3 receptor blocker 2-APB (100 μM) could also block PrRPs-induced LH secretion and mRNA expression in grass carp pituitary cells, respectively (**Figures 6**, **7**). Finally, the VSCC inhibitor nifedipine (10 μM), CaM antagonist calmidazolium (1 μM) or CaMk-II blocker KN62 (5 μM) could also block PrRPs-induced LH secretion and mRNA expression in grass carp pituitary cells, which suggested that Ca^2+^/CaM/CaMK-II pathways could be involved in the regulation of pituitary secretion and mRNA expression by PrRPs (**Figures 6**, **7**).

### Receptor Specificity for the Regulation LHβ Promoter Activity by PrRPs

**Figure 8:**
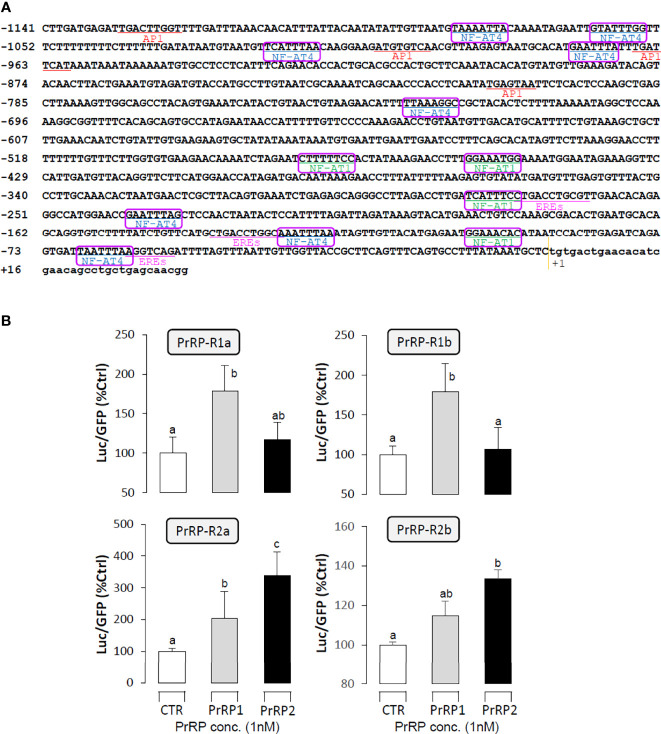
Receptor specificity for the regulation of LHβ promoter activity by PrRPs in HEK-293T cells. **(A)** The LHβ promoter sequence was used to predict putative binding sites by the JASPAR database and Signal Scan. **(B)** LH promoter and PrRP receptor plasmids were transiently co-transfected into HEK-293T cells as well. In the parallel experiment, GFP and renilla luciferase were used as the internal control. Data presented are expressed as mean ± SEM, and the differences between groups were significant at P-value < 0.05 by labeling diverse letters.

The LHβ promoter, which contained 1141bp from upstream to the transcription start point, was used to predict the putative binding sites that incorporated 3 half estrogen response elements (EREs), 4 AP-1 binding sites, and 12 NFAT-luciferase elements. (**Figure 8A**). To verify the direct regulation of PrRPs by activating their specific GPCRs and cause LHβ mRNA expression, the LHβ promoter and PrRP receptor plasmids were transiently co-transfected into HEK-293T cells, then the promoter assays were used to detect luciferase activity. The result showed that PrRP1 and PrRP2 could both dramatically elevate LHβ promoter activity through activating the four PrPR-Rs (**Figure 8B**). Interestingly, these stimulatory effects by PrPR1 and PrRP2 showed a different receptor selectivity. PrRP1 preferentially activated PrRP-R1a and PrRP-R1b to induce LHβ promoter activity. However, PrRP2 tended to select PrRP-R2a and PrRP-R2b to stimulate LHβ promoter activity (**Figure 8B**). In addition, PrRP2-activating PrRP-R2a was found to be the most potent in triggering the stimulating effects on LHβ promoter activity (**Figure 8B**). These results indicated that PrRP1 and PrRP2 could both be directly involved in the regulation of LHβ expression by activating their specific receptors.

### *In Vivo* Test of PrRPs in Grass Carp

**Figure 9:**
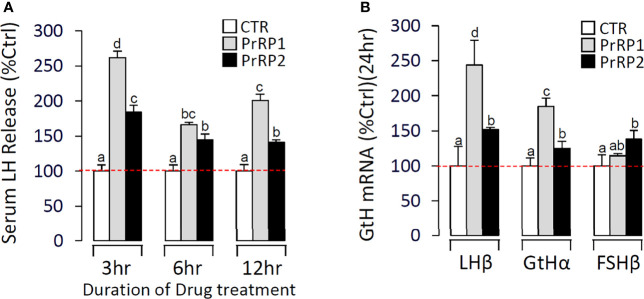
*In vivo* test of PrRPs in grass carp. **(A)** In the time-course test, blood was collected at 3 h, 6 h, and 12 h and analyzed for serum LH secretion which was induced by PrRPs. **(B)** After a single-dose IP injection of PrRPs for 24 h, the pituitaries were taken to detect the transcript level of LHβ, GtHα, and FSHβ. Data presented are expressed as mean ± SEM, and the differences between groups were significant at P-value < 0.05 by labeling diverse letters.

Using grass carp as the model, PrRP1 and PrRP2 were intraperitoneally injected with a specified dose (100 ng/g body weight) dissolved in saline (0.7% NaCl). After 24 h treatment, PrRP1 and PrRP2 could both significantly induce pituitary LHβ and GtHα mRNA expression, whereas only a slight increase for FSHβ mRNA expression was detected in grass carp pituitary (**Figure 9B**). Besides, in the parallel experiments, PrRP1 injection caused an approximately 2.6-fold increase in the serum LH release level within 3 h, followed by the LH secretion had a declining fluctuation to 2-fold within 6-12 h (**Figure 9A**). PrRP2 could also evoke a 1.8-fold increase of serum LH release at 3 h (**Figure 9A**).

### Effect of Estradiol on PrRPs and PrRP-Rs mRNA Expression in Grass Carp Brain Cells

**Figure 10:**
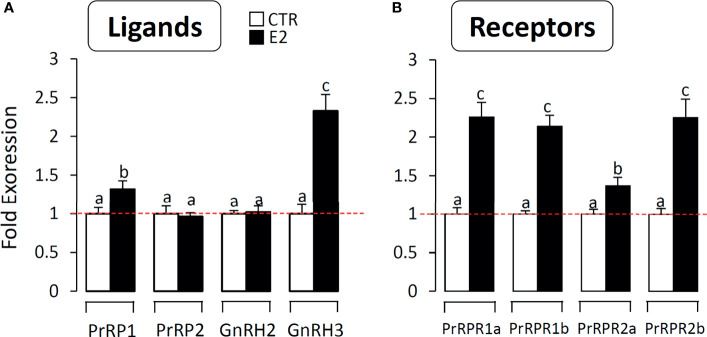
The effect of E2 on PrRPs and PrRP-Rs mRNA expression in grass carp brain cells. In this study, E2 (final concentration 1 μM) was used to incubate grass carp brain cells for 24 h. After drug treatment, total RNA were extracted for RT-PCR to detect the PrRPs, GnRH2, GnRH3 **(A)** and PrRP-Rs **(B)** mRNA expression. Data presented are expressed as mean ± SEM, and the differences between groups were significant at P-value < 0.05 by labelling diverse letters.

To further confirm the involvement of the PrRP system in reproduction, we detected the effect of estradiol on PrRPs and PrRP-Rs mRNA expression in primary cultured grass carp brain cells. The results indicated that estradiol could significantly induce PrRP1 and particularly GnRH3 mRNA expression in grass carp brain cells (**Figure 10A**). In parallel, four PrRP-Rs mRNA expression could also be significantly induced by E2 treatment (**Figure 10B**), which suggested the involvement of the piscine PrRP system in the regulation of reproduction.

### The Effect of Feeding on PrRPs mRNA Expression and Blood Glucose Level

**Figure 11:**
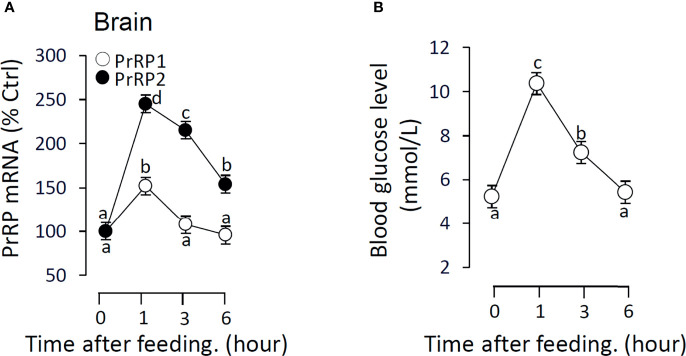
The effect of food intake on brain PrRPs mRNA expression and blood glucose level in grass carp. Grass carp entrained with a one-meal-per-day feeding schedule (with six fish per group) was provided with fish feed at 9:00 Am (taken as 0 hour). **(A)** The brain was collected at specific points in time (0, 1, 3, 6 h) and total RNA were extracted to measure the PrRPs mRNA expression by real-time PCR. **(B)** The blood glucose level was detected using the tail cutting process by a blood glucose meter at 0, 1, 3, 6 h after feeding. In this experiment, β-actin was used as an internal control in RT-PCR. Data presented are expressed as mean ± SEM, and the differences between groups were significant at P-value < 0.05 by labeling diverse letters.

To verify the relationship between feeding and PrRPs expression level, we detected the postprandial changes on brain PrRPs mRNA expression. The results showed that food intake could significantly induce brain PrRP2 mRNA expression with a peak value at 1 h but rapidly descent followed at 3 and 6 h (**Figure 11A**). In parallel, the transcript level of PrRP1 could also be significantly increased in 1 hour after food intake (**Figure 11A**). Besides, the postprandial blood glucose level observably increased within 1 hour, whereas this rise gradually vanished later and returned to initial blood glucose levels within 6 hours (**Figure 11B**).

## Discussion

In mammals, PrRP, which could encode two peptides of different lengths named PrRP20 and PrRP31, has been initially found to stimulate PRL release (11). However, in nonmammalian vertebrate species, another novel bioactive peptide has been isolated from crucian carp, namely Carassius RFamide (C-RFa) (41). This 20-amino acid peptide, as a homologue of PrRP20, was subsequently found in chicken (42), frog, zebrafish (43), tilapia (44), and chum salmon (45). This form of PrRP is highly conserved and shows almost 100% similarity in identified species. In our present study, two forms of PrRP (C-RFa was named PrRP1 and PrRP20 was named PrRP2 in this paper) have also been identified in grass carp. During evolution, all extant vertebrates have experienced two rounds (2R) of whole genome duplication (WGD) in their evolutionary process (46). Besides, it has been reported that the third round (3R) of WGD occurred in the stem lineage of ray-finned (actinopterygian) fishes (47, 48). In this process, most duplicated genes were secondarily lost after genome duplication (49), and only some duplicated genes could spawn new functions and survive (50). Therefore, we inferred that an ancestral R-Fa sequence motif gene produced C-RFa and PrRP20 isoforms by 1R and 2R WGD, so both two isoforms were found in amphibian, reptile, and avian. Due to the gene loss during evolution in mammalian vertebrates, such as rats and humans, only the PrRP20 subtype was found while C-RFa experienced a degenerate process and was definitively lost. In fish, we speculated four expected subtypes of PrRP were obtained based on 3R WGD. However, putative PrRP1b and PrRP2b isoforms were lost, indicating that only PrRP1, PrRP2 played a role (**Supplementary Figure S12**).

The human PrRP-receptor was initially isolated from the pituitary as an orphan seven-transmembrane-domain receptor (11). This is nearly identical to GPR10, which was cloned as a candidate G-coupled receptor named UHR-1 from rat hypothalamus in a previous study (51). After that, based on the sequence homology of mammal-PrRP, three and four receptors have been predicted and cloned in chicken and zebrafish, respectively (43, 52). In grass carp, similar to zebrafish, four receptors (that is PrRP-R1a, PrRP-R1b, PrRP-R2a, PrRP-R2b) were cloned, verifying the conjecture that 3R WGD occurred in teleost. Although the receptors have been cloned in teleost, the rank order of ligand selectivity based on PrRPs has not been fully identified. In the present study, according to the ligand-receptor selectivity test, we have confirmed that newly cloned grass carp PrRPRs and hPrRPR could both be functionally coupled with PKC-dependent signaling mechanisms. Comparing the respective ED_50_ of PrRPR1 indicated that the grass carp PrRPR1a and PrRPR1b could both exhibit a rank order of PrRP1 > PrRP2 > hPrRP for receptor activation. In contrast, grass carp PrRPR2a and PrRPR2b could both show a rank order of PrRP2 > PrRP1 > hPrRP for receptor activation. The result proved that PrRP showed an evident receptor preference in accomplishing functional expression. Due to the 3R WGD, an extra GPR10 (hPrRPR) counterpart was obtained, together with another PrRPR2, both showed a high affinity with PrRP2, which was deemed as the homologous isoform of hPrRP. PrRP-R1 can function as the specific receptors for PrRP1. Interestingly, hPrRP could merely activate grass carp PrRP-R1b when it showed low activation capacity to other grass carp PrRP-Rs. In addition, we found that PrRP1, PrRP2, and hPrRP could all activate hPrRPR, which was similar to the result in chicken (43). We deduced that the conserved amino acid sequence of PrRP receptors in the transmembrane region after sequence alignment was the key binding site where ligands combined corresponding receptors (**Supplementary Figure S13**).

Our main aim was to test the involvement of the PrRP/PrRP-R system in reproduction. According to the RT-PCR assay, PrRPs and PrRP-Rs were highly detected in the hypothalamus and pituitary, suggesting the potential pituitary function induced by PrRPs in the HPG axis. In mammals, previous studies have reported that IP injection of PrRP could significantly stimulate serum LH release in rats (20), but little is known about the mechanism of PrRP-induced LH expression. In the present study, to verify the function of PrRP on reproduction, IP injection was performed in grass carp and we found that both PrRP1 and PrRP2 could significantly induce serum LH release and pituitary LHβ mRNA expression. These results suggested that PrRP could also play an important role in reproduction in teleost. Unlike mammals, the localized circulatory system namely the median eminence was inexistent in teleost (53). Therefore, there is a peculiar structure that axons of hypothalamic neurons project directly to post-synaptic sites in the teleost pituitary (54). To verify whether PrRP could directly act on the pituitary to induce LH secretion and synthesis, using grass carp primary cultured pituitary cells as a model, we further found PrRP1 and PrRP2 could time- and dose-dependently stimulate LHβ mRNA expression and LH release *via* the AC/PKA, PLC/IP3/PKC cascades and Ca2+/CaM/CaMK-II cascade. Finally, clear evidence exists regarding the important role estradiol plays during the period of reproduction in fish. A recent study indicated that estradiol treatment of prepubertal zebrafish enhanced the expression of the key genes involved in reproduction (GnRH3, Kiss2, and Tac3) (55). In the present study, we also found that estradiol could significantly stimulate PrRP1, PrRPR1, and PrRPR2 mRNA expression in primary culture grass carp hypothalamus cells. These results further confirmed that PrRP systems were involved in the reproductive process.

Our next aim was to test the involvement of the PrRP system in mediating energy balance and reproduction. Reproduction is vital to the sustainable survival of species, but this process needs huge energy expenditure. Therefore, the close connection between energy balance and reproduction has been well documented in mammals (22, 23). Energy balance is maintained by a process that controls food consumption, energy expenditure, and energy storage. The hypothalamus plays an important role in the regulation of both energy balance and reproduction. The neuroendocrine mechanisms responsible for the association between energy balance and fertility are represented by metabolic hormones and neuropeptides that affect the hypothalamic center controlling the expression and release of GnRH (56). Similar to GnRH, it has been identified that the PrRP neurons involved in the regulation of food intake and energy homeostasis (1, 9). In mammals, PrRP administered centrally reduced food intake (2), and fasting rats showed a reduction in the expression of PrRP mRNA (2, 9), in which PrRP interacted with leptin and corticotropin-releasing hormone (CRH) to play an anorectic role (57). In goldfish, both IP and ICV injection of PrRP could reduce food intake, and PrRP mRNA expression increased after feeding (10). In our study, postprandial PrRP1 and PrRP2 mRNA significantly increase in a short time after initial feeding, indicating that PrRP acts as a transient anorexigenic factor in grass carp. Feeding could induce a short-term rise in blood glucose, which was observed in postprandial grass carp. In mice, the PrRP-KO category showed a slight increase in glucose concentrations compared with WT groups of mice that also revealed significant glucose intolerance (58). PrRP-treatment could effectively improve glucose tolerance (59, 60) but intracerebroventricular (ICV) injections of PrRP showed no effect on blood glucose concentration in chicks (61). These results indicate that PrRP plays a role as an endogenic factor to interact with blood glucose, and PrRP could not directly affect the blood glucose level.

**Figure 12:**
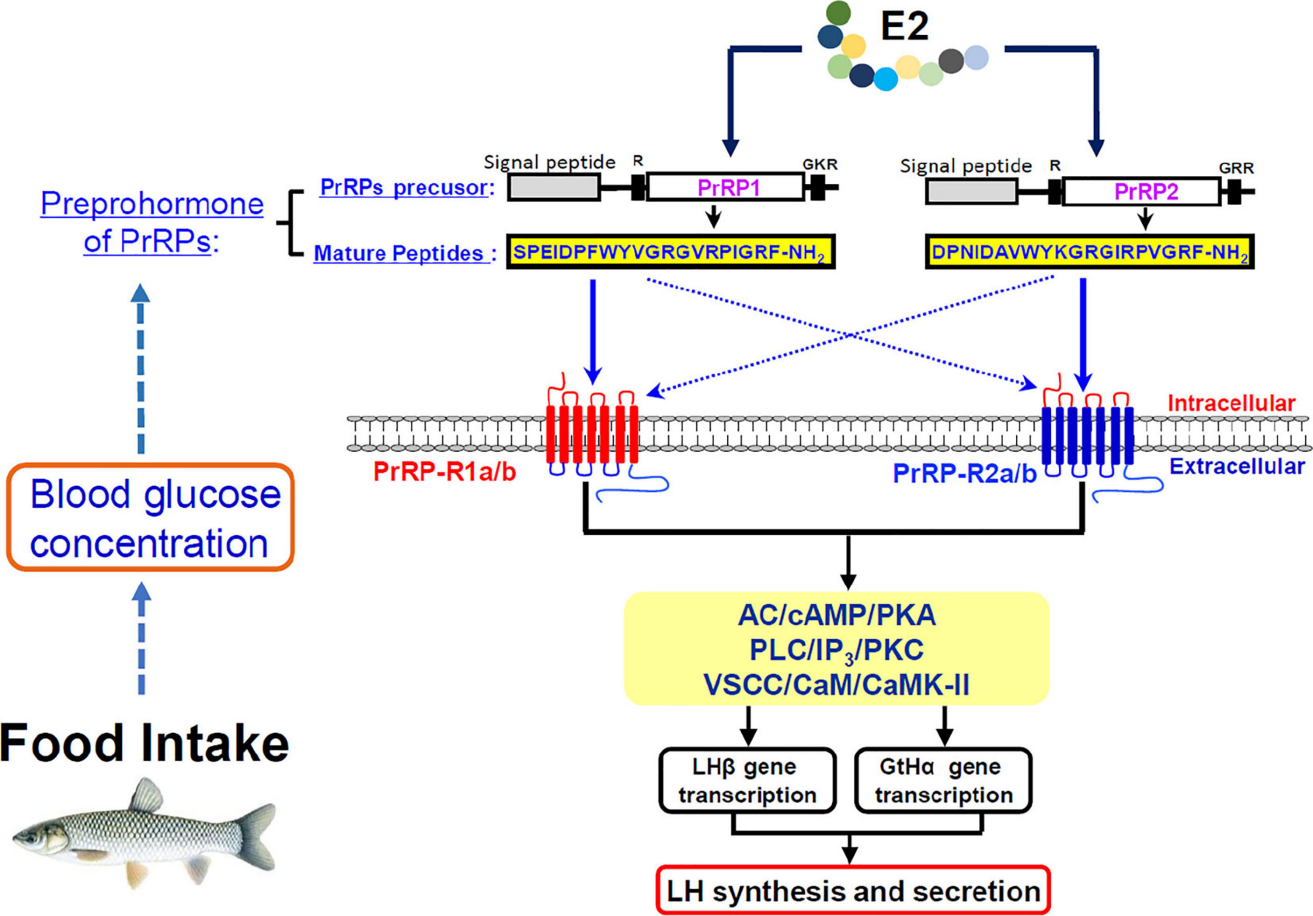
Working model of PrRP mediated feeding and reproduction in grass carp. Food intake could observably induce PrRP mRNA expression and blood glucose level. Two PrRP ligands and four PrRP receptors were cloned from grass carp brain, and ligand-receptor selectivity showed that PrRP-R1 can function as the receptors for PrRP1, whereas PrRP-R2 were receptors specific to PrRP2. Besides, PrRP1 and PrRP2 can stimulate LHβ, GtHα mRNA expression and LH secretion in grass carp pituitary *via* AC/cAMP/PKA, PLC/IP3/PKC and Ca2+/CaM/CaMK-II pathways *in vitro*, meanwhile IP injection PrRPs could induce LHβ, GtHα mRNA expression, and LH secretion as well *in vivo*. Finally, estrogen treatment could elicit increases in PrRPs and PrPR receptors mRNA expression in primary cultured brain cells.

In summary, the two PrRP ligands (PrRP1 and PrRP2) and four PrRP receptors (including PrRP-R1a, PrRP-R1b, PrRP-R2a, PrRP-R2b) were cloned from grass carp brain and pituitary. Then ligand-receptor selectivity showed that PrRP1 and PrRP2 had different degrees of affinity to the four receptors in grass carp, for example, PrRP-R1a/b can function as the receptors for PrRP1, whereas PrRP-R2a/b were the specific receptors for PrRP2. Tissue distribution indicated that both PrRPs and PrRP-Rs were highly detected in the hypothalamus-pituitary-gonad axis and intestine, suggesting latent function on food intake and reproduction. IP injection PrRP1 and PrRP2 could both induce serum LH release and pituitary LHβ and GtHα mRNA expression. In addition, using primary pituitary cells as a model, we further found that PrRP1 and PrRP2 could both significantly induce pituitary LH secretion and mRNA expression mediated by AC/PKA, PLC/IP3/PKC, and Ca^2+^/CaM/CaMK-II pathways. E2 treatment could significantly induce *Prrps* and *Prrp* receptors mRNA expression in primary cultured brain cells, which confirmed that the PrRP/PrRPR system was involved in the neuroendocrine control of fish reproduction. Finally, the feeding experiment proved that food intake could notably induce PrRP mRNA expression and blood glucose level, suggesting that PrRP should be an anorexigenic peptide in teleost. This study not only proved that the PrRP system could involve reproductive regulation in the teleost, it also indicated that they could mediate energy balance and reproduction (**Figure 12**).

## Data Availability Statement

The datasets presented in this study can be found in online repositories. The names of the repository/repositories and accession number(s) can be found in the article/**Supplementary Material**.

## Ethics Statement

The animal study was reviewed and approved by the Huazhong Agricultural University Administrative Panel for Laboratory Animal Care (Ethical Approval No. HBAC20091138; Date: 15 November 2009).

## Author Contributions

Data curation, CX and XFQ. Formal analysis, LZ. Funding acquisition, GH and ZY. Investigation, XS, TC, and YX. Methodology, RD, WL, YY, CX, and XQ. Resources, XS and CX. Software, LZ and XS. Supervision, GH and ZY. Writing – original draft, CX. Writing – review and editing, GH. All authors contributed to the article and approved the submitted version.

## Funding

Funding support was provided by the National Key R&D Program of China (2018YFD0900205 to ZY), the China Postdoctoral Science Foundation (2019M662747 to GH), Fundamental Research Funds for the Central Universities (2662019PY006 to GF), and Hubei Province Postdoctoral Science Foundation (237934 to GH).

## Conflict of Interest

The authors declare that the research was conducted in the absence of any commercial or financial relationships that could be construed as a potential conflict of interest.

## Publisher’s Note

All claims expressed in this article are solely those of the authors and do not necessarily represent those of their affiliated organizations, or those of the publisher, the editors and the reviewers. Any product that may be evaluated in this article, or claim that may be made by its manufacturer, is not guaranteed or endorsed by the publisher.

## Abbreviations

AC, adenylate cyclase; cAMP, cyclic adenosine monophosphate; PrRP, Prolactin-releasing peptide; PrRPR, Prolactin-releasing peptide receptor; IP, intraperitoneal; LH, luteinizing hormone; GtHα, gonadotropin subunit alpha; PKA, protein kinase A; PLC, phospholipase C; IP3, inositol trisphosphate PKC, protein kinase C; CaM, calmodulin; CaMK, calmodulin kinase; GPR, G protein coupled receptor; ICV, intra-cerebroventricular; PRL, prolactin; HPG, hypothalamus-pituitary-gonad; GnRH, gonadotropin-releasing hormone; ORF, open reading frame; RT-PCR, reverse transcription-polymerase chain reaction; NFAT, nuclear factor of activated T cells; FSHβ, follicle-stimulating hormone beta; TMD, transmembrane domain; C-RFa, Carassius RFamide; WGD, whole genome duplication.
