# Supplementary figures and images for: Reproductive Regulation of PrRPs in Teleost: The Link Between Feeding and Reproduction
Source: Front Endocrinol (Lausanne). 2021 Nov 3;12:762826. doi: 10.3389/fendo.2021.762826 (PMC8595397; doi:10.3389/fendo.2021.762826)

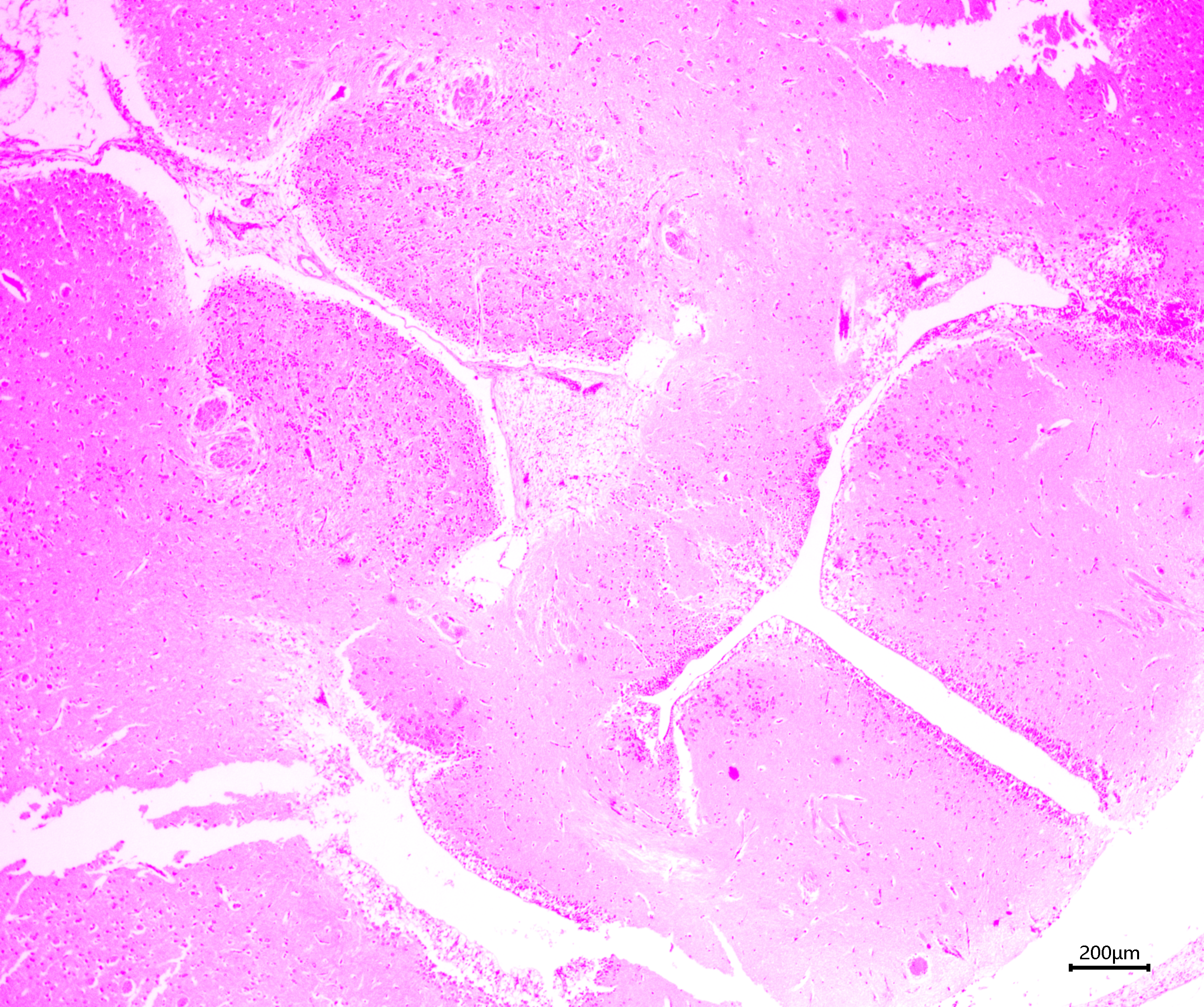

Supplement: Supplementary file 1 [file DataSheet_1.zip › Raw data/Figure1/Figure1σàìτû1⁄2ΦìoσàëσÅèHEμƒôΦë▓/HE/22.tif]

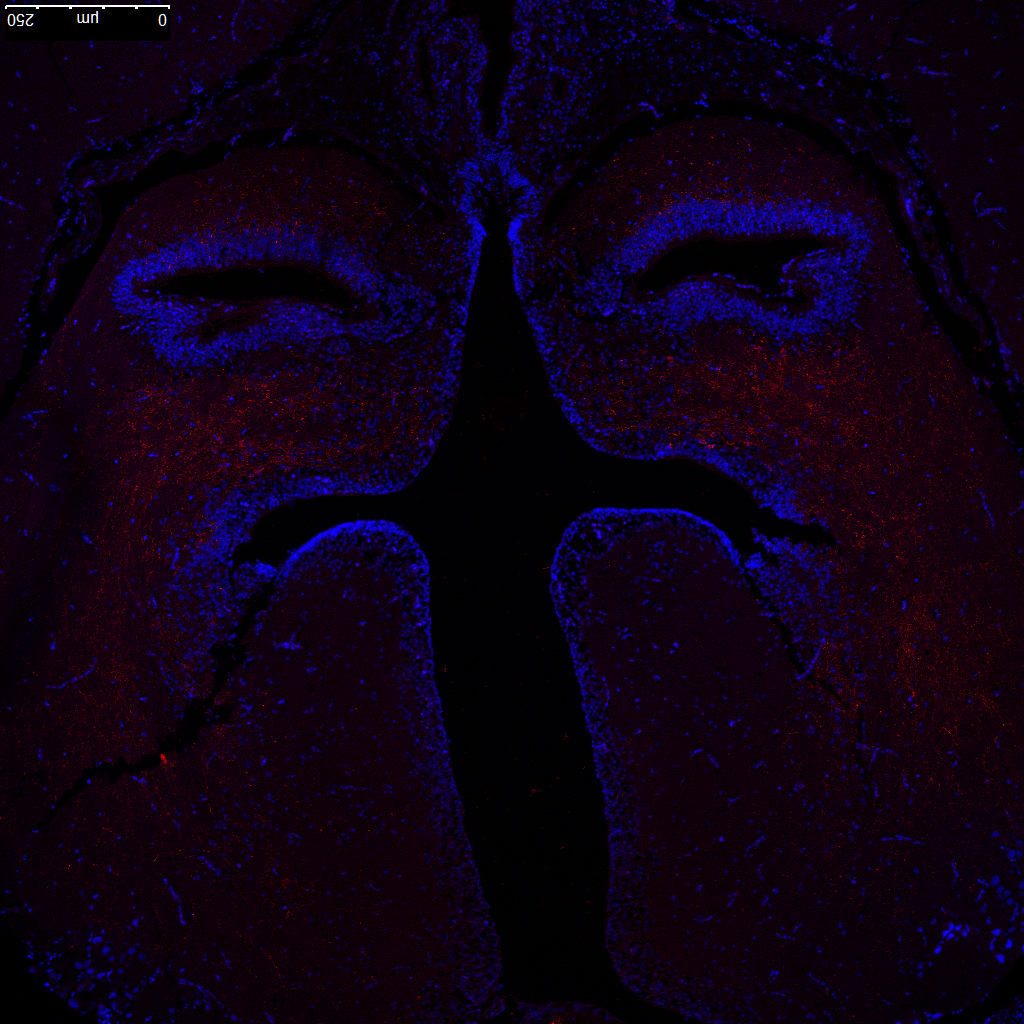

Supplement: Supplementary file 1 [file DataSheet_1.zip › Raw data/Figure1/Figure1σàìτû1⁄2ΦìoσàëσÅèHEμƒôΦë▓/σàìτû1⁄2Φìoσàë/prrp1 1.800 gc brain 1.tif]

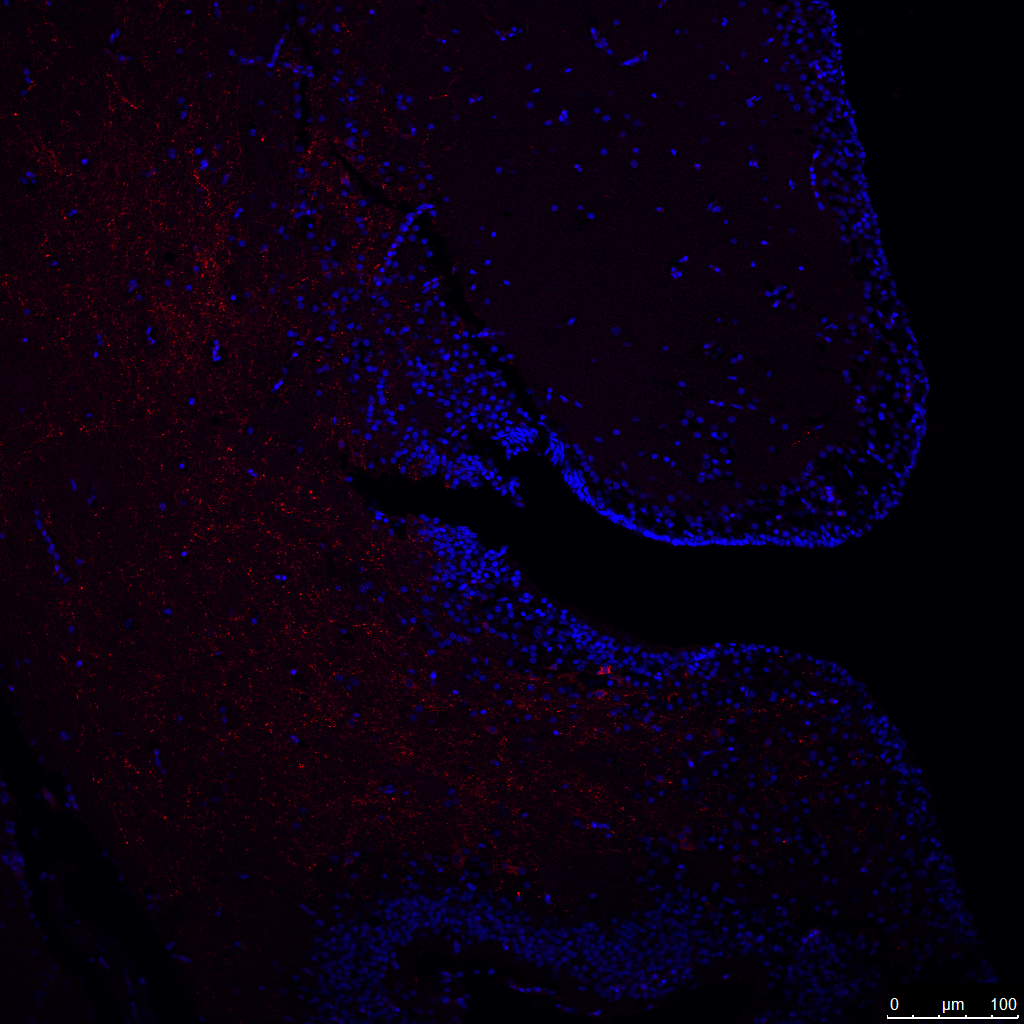

Supplement: Supplementary file 1 [file DataSheet_1.zip › Raw data/Figure1/Figure1σàìτû1⁄2ΦìoσàëσÅèHEμƒôΦë▓/σàìτû1⁄2Φìoσàë/prrp1 1.800 gc brain 3.tif]

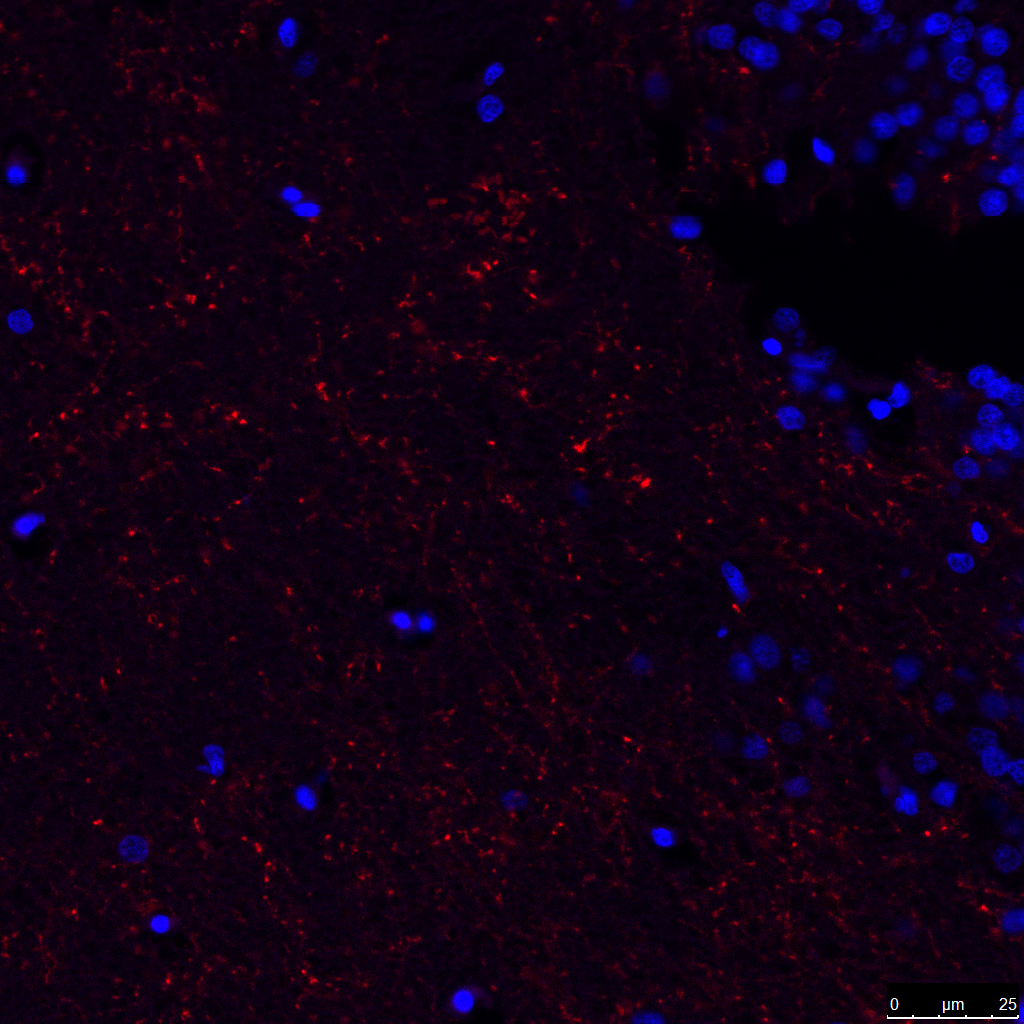

Supplement: Supplementary file 1 [file DataSheet_1.zip › Raw data/Figure1/Figure1σàìτû1⁄2ΦìoσàëσÅèHEμƒôΦë▓/σàìτû1⁄2Φìoσàë/prrp1 1.800 gc brain 5.tif]

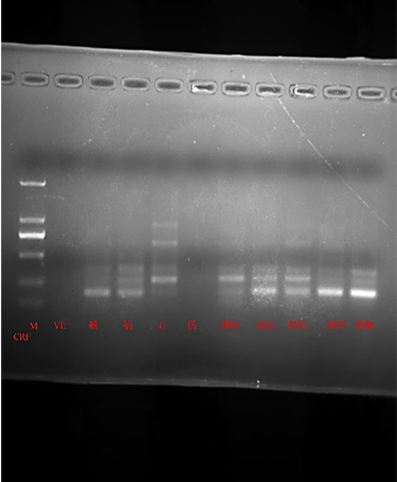

Supplement: Supplementary file 2 [file DataSheet_2.zip › Figure1/Figure1 τ╗äτ╗çΦí¿Φ╛╛σêåμ₧ÉΦâ╢σ¢╛/PrRP1 FigureC Bottom.png]

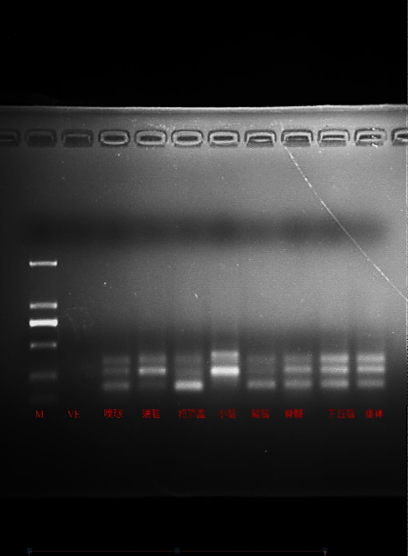

Supplement: Supplementary file 2 [file DataSheet_2.zip › Figure1/Figure1 τ╗äτ╗çΦí¿Φ╛╛σêåμ₧ÉΦâ╢σ¢╛/PrRP1 FigureC Top.png]

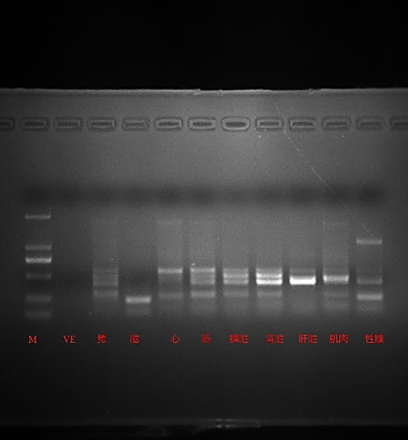

Supplement: Supplementary file 2 [file DataSheet_2.zip › Figure1/Figure1 τ╗äτ╗çΦí¿Φ╛╛σêåμ₧ÉΦâ╢σ¢╛/PrRP2 FigureC Bottom.png]

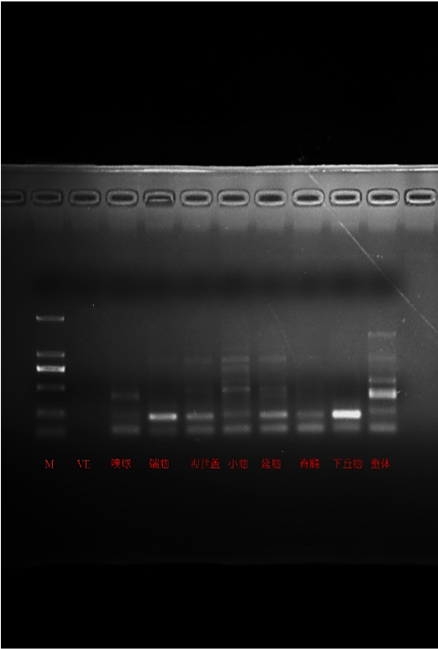

Supplement: Supplementary file 2 [file DataSheet_2.zip › Figure1/Figure1 τ╗äτ╗çΦí¿Φ╛╛σêåμ₧ÉΦâ╢σ¢╛/PrRP2 FigureC Top.png]

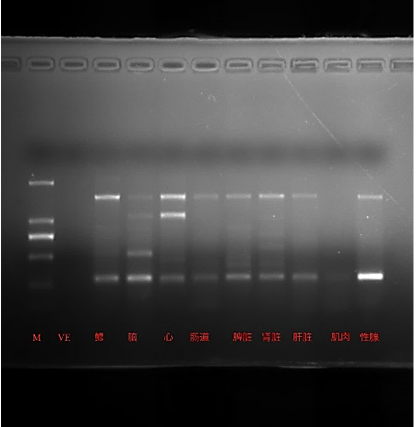

Supplement: Supplementary file 2 [file DataSheet_2.zip › Figure1/Figure1 τ╗äτ╗çΦí¿Φ╛╛σêåμ₧ÉΦâ╢σ¢╛/PrRPR1a FigureC Bottom.png]

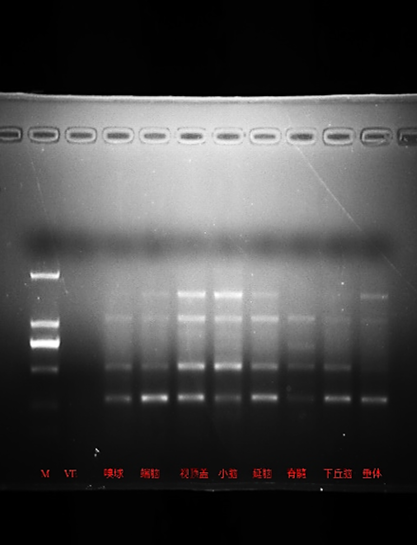

Supplement: Supplementary file 2 [file DataSheet_2.zip › Figure1/Figure1 τ╗äτ╗çΦí¿Φ╛╛σêåμ₧ÉΦâ╢σ¢╛/PrRPR1a FigureC Top.png]

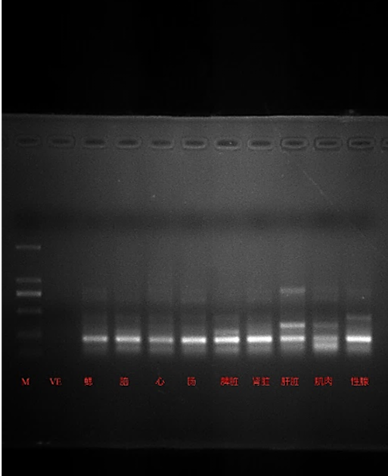

Supplement: Supplementary file 2 [file DataSheet_2.zip › Figure1/Figure1 τ╗äτ╗çΦí¿Φ╛╛σêåμ₧ÉΦâ╢σ¢╛/PrRPR1b FigureC Bottom.png]

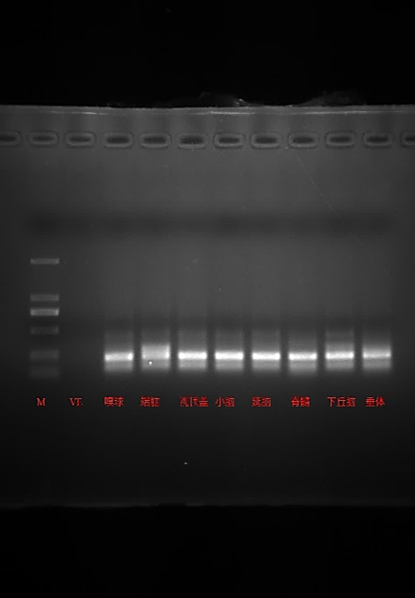

Supplement: Supplementary file 2 [file DataSheet_2.zip › Figure1/Figure1 τ╗äτ╗çΦí¿Φ╛╛σêåμ₧ÉΦâ╢σ¢╛/PrRPR1b FigureC Top.png]

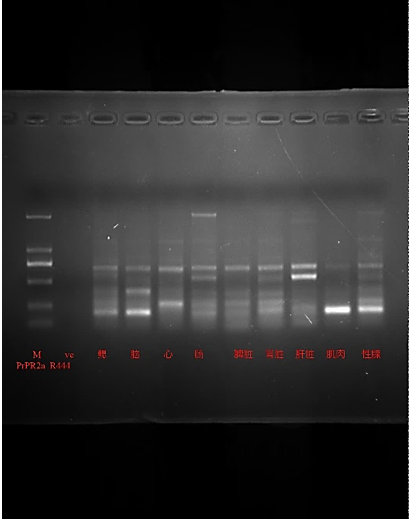

Supplement: Supplementary file 2 [file DataSheet_2.zip › Figure1/Figure1 τ╗äτ╗çΦí¿Φ╛╛σêåμ₧ÉΦâ╢σ¢╛/PrRPR2a FigureC Bottom.png]

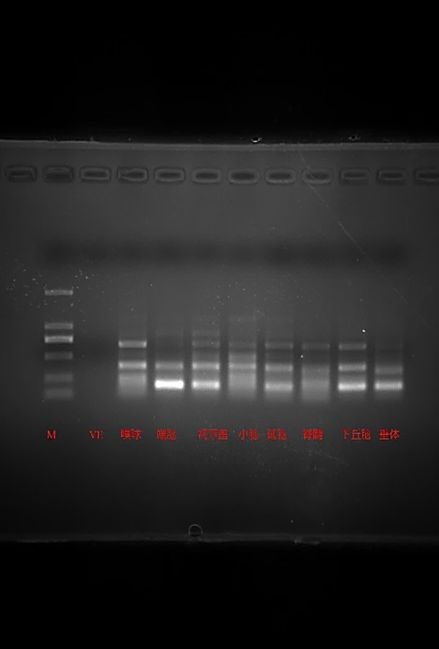

Supplement: Supplementary file 2 [file DataSheet_2.zip › Figure1/Figure1 τ╗äτ╗çΦí¿Φ╛╛σêåμ₧ÉΦâ╢σ¢╛/PrRPR2a FigureC Top.png]

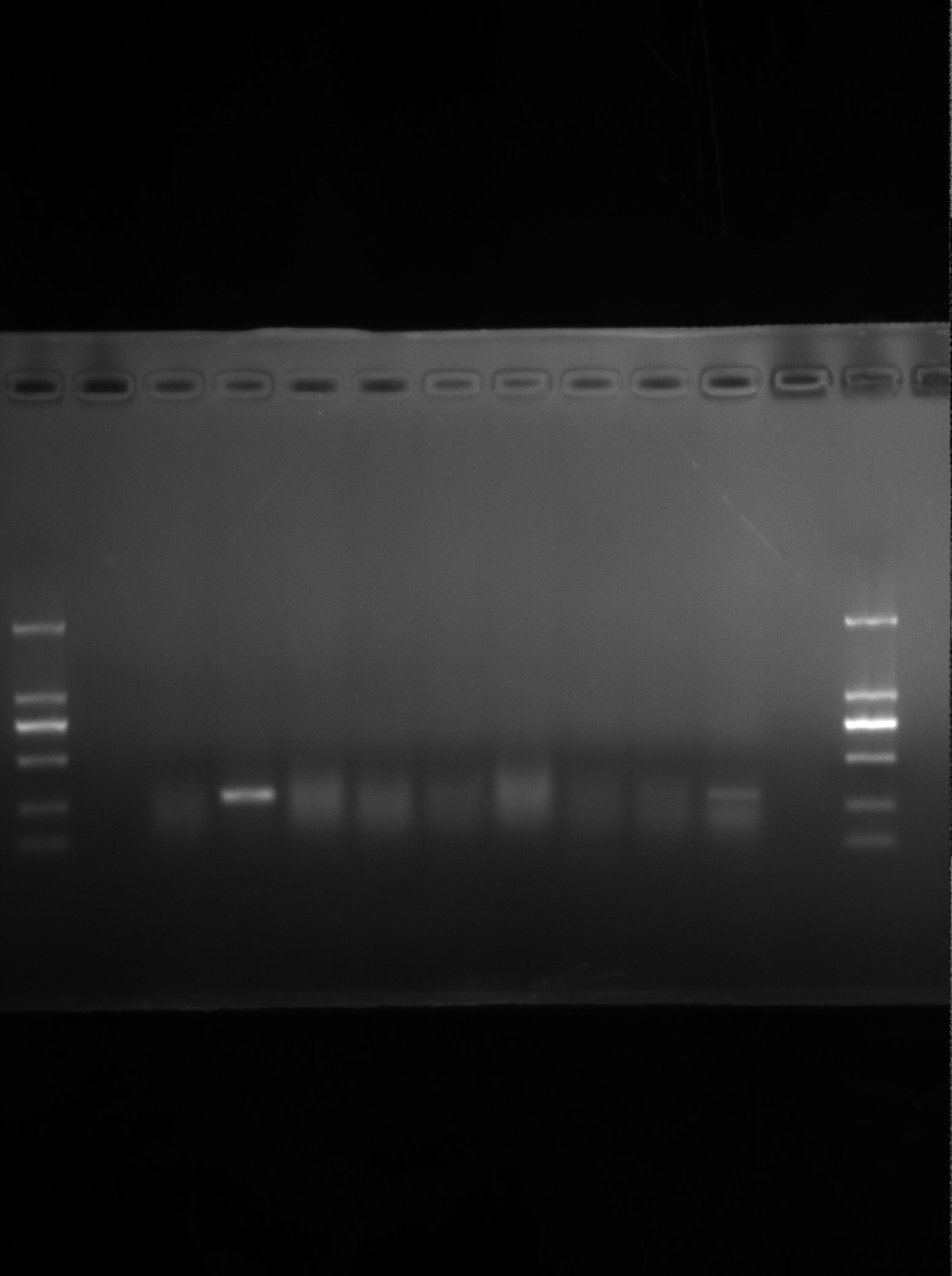

Supplement: Supplementary file 2 [file DataSheet_2.zip › Figure1/Figure1 τ╗äτ╗çΦí¿Φ╛╛σêåμ₧ÉΦâ╢σ¢╛/PrRPR2b FigureC Bottom.png]

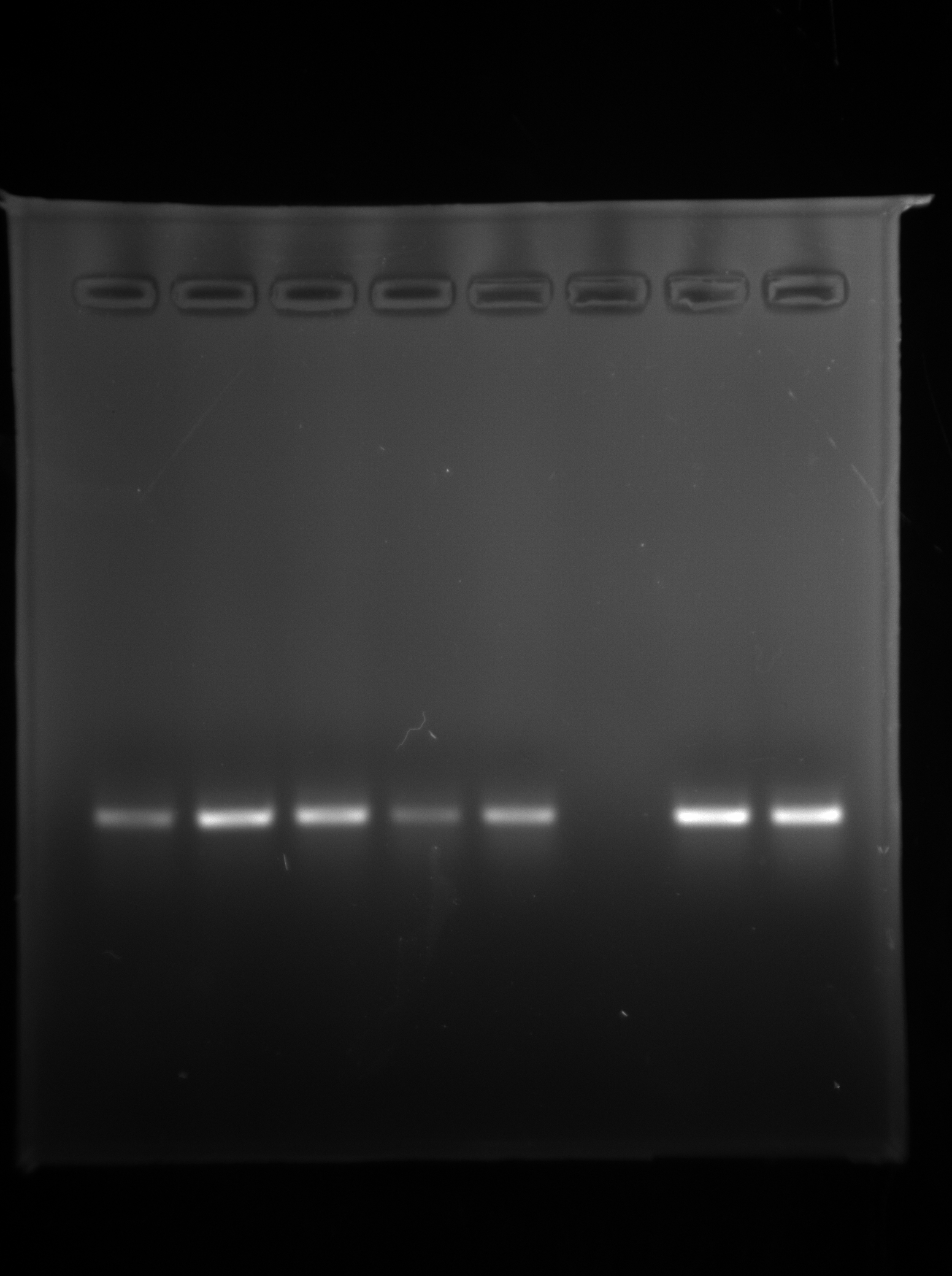

Supplement: Supplementary file 2 [file DataSheet_2.zip › Figure1/Figure1 τ╗äτ╗çΦí¿Φ╛╛σêåμ₧ÉΦâ╢σ¢╛/PrRPR2bFigureC Top.png]

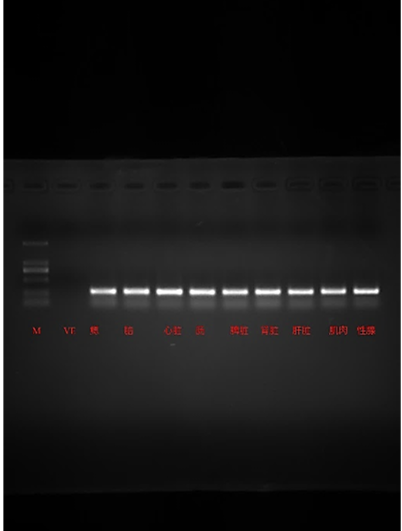

Supplement: Supplementary file 2 [file DataSheet_2.zip › Figure1/Figure1 τ╗äτ╗çΦí¿Φ╛╛σêåμ₧ÉΦâ╢σ¢╛/╬▓-actin FigureC Bottom.png]

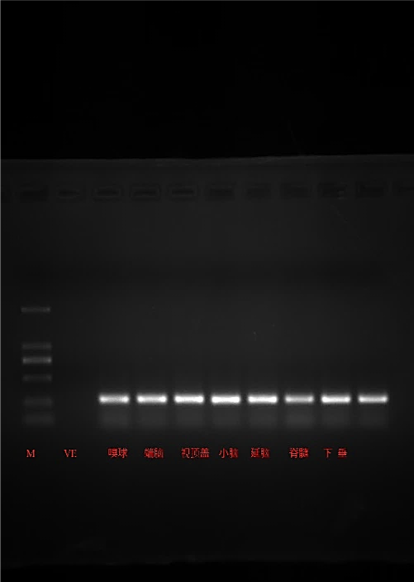

Supplement: Supplementary file 2 [file DataSheet_2.zip › Figure1/Figure1 τ╗äτ╗çΦí¿Φ╛╛σêåμ₧ÉΦâ╢σ¢╛/╬▓-actin FigureC Top.png]

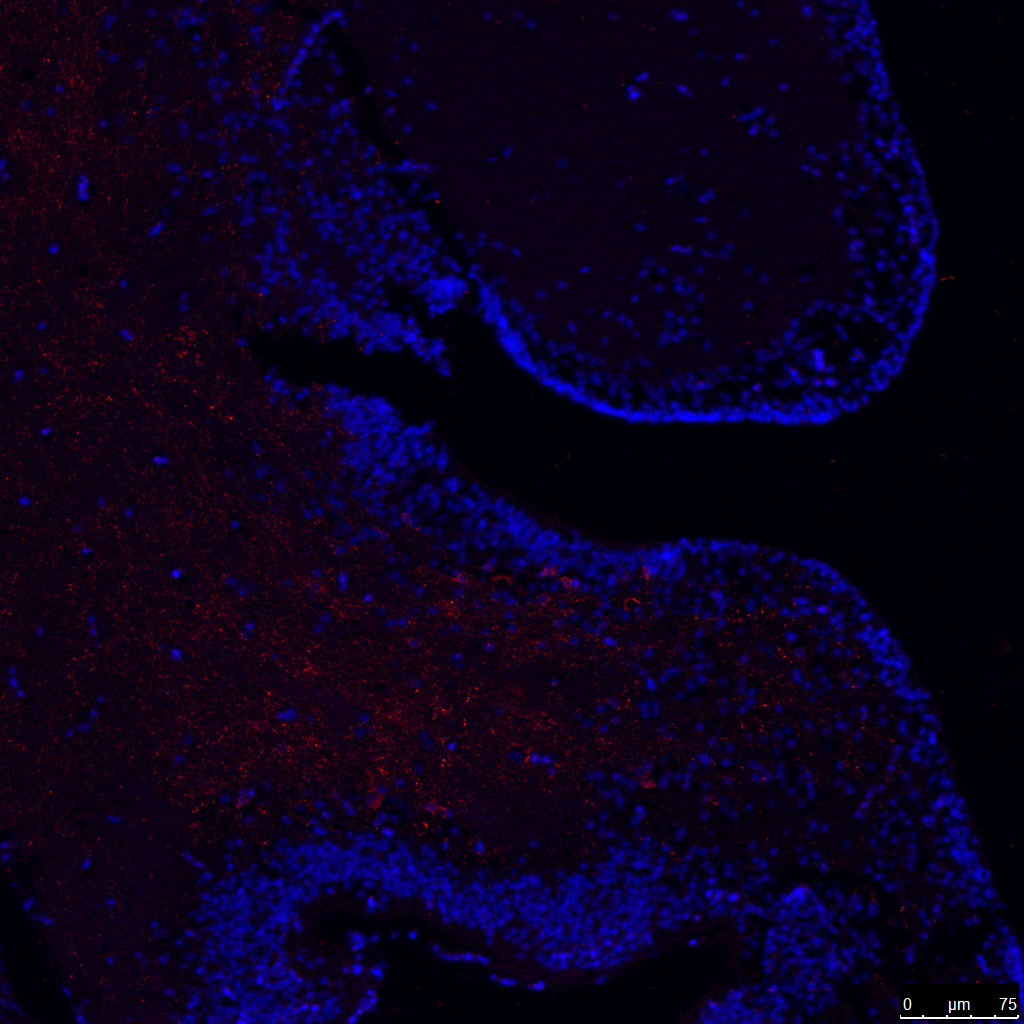

Supplement: Supplementary file 2 [file DataSheet_2.zip › Figure1/Figure1σàìτû1⁄2ΦìoσàëσÅèHEμƒôΦë▓/σàìτû1⁄2Φìoσàë/Figure F.tif]

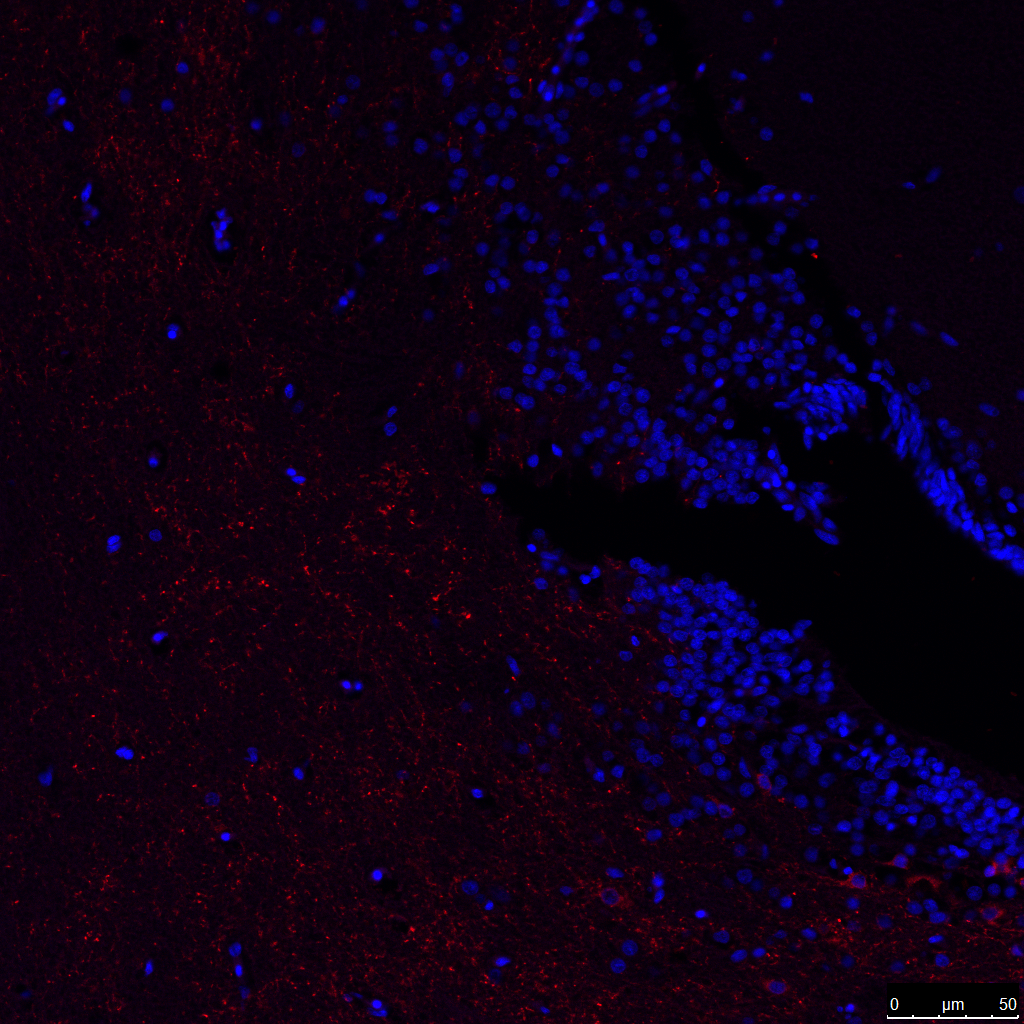

Supplement: Supplementary file 2 [file DataSheet_2.zip › Figure1/Figure1σàìτû1⁄2ΦìoσàëσÅèHEμƒôΦë▓/σàìτû1⁄2Φìoσàë/Figure G.tif]
