# Supplementary material for: Reproductive Regulation of PrRPs in Teleost: The Link Between Feeding and Reproduction
Source: Front Endocrinol (Lausanne). 2021 Nov 3;12:762826. doi: 10.3389/fendo.2021.762826 (PMC8595397; doi:10.3389/fendo.2021.762826)
Supplement: Supplementary file 3 [file DataSheet_3.pdf]

SUPPLEMENTARY INFORMATION

Reproductive regulation of PrRPs in teleost: the link between feeding and reproduction

Chuanhui Xia#, Xiangfeng Qin#, Lingling Zhou, Xuetao Shi, Tianyi Cai, Yunyi Xie, Qiongyao Hu, Shaohua Xu, Zhan Yin*, Guangfu Hu*

[1]College of Fisheries, Hubei Province Engineering Laboratory for Pond Aquaculture, Huazhong Agricultural University, Wuhan, 430070, China

[2]State Key Laboratory of Freshwater Ecology and Biotechnology, Institute of Hydrobiology, Chinese Academy of Sciences, Wuhan 430072, China

Supplementary Table S1 | Background information for medicine used in *in vitro* experiments

name	function	Sales company	article number
H89	PKA inhibitor	Calbiochem	371963
2-APB	IP3 receptor retardant	Calbiochem	100065
KN62	CaMK-II retardant	Calbiochem	422706
U-73122	PLC inactivator	Sigma	U6756
GF109203X	PKC inhibitor	Sigma	B6292
Nifedipine	VSCC retardant	Sigma	N7634
Calmidazolium	CaM antagonist	RBI	C-100
MDL12330A	AC inhibitor	Merck	444200

Supplementary Table S2 | Primer sequences and PCR conditions for open read regions (ORF) for selected gene targets and LHβ promoter in grass carp

Gene	Forward primer	Reverse primer	Annealing Tm (℃)	Product size (bp)
PrRP1 (S)	AGATCTGCTGATGTAGTGCG	TCACGGTAACCAGTCTGAGTC	56	537
PrRP2 (D)	ATGGTTGTAAAGCTGTGTGT	TTACCCATTGATGCCATATG	58	297
PrPR-R1a	CATGACGGACATGGACTATCT	TCAATCTTGTGAAATCTTACT	58	1117
PrPR-R1b	ATGGATCCTCTTCTGGAACAA	TCAGAGGCAGCTGTCAGGGA	56	1134
PrPR-R2a	ATGGATGGCAGTGGTGGTGAATGG	TCAGAGAACCACGCTAGCAGT	56	1113
PrPR-R2b	ATGGAGGGCTCTGGCTGGC	TCATAGTACGACGCTTGCCGTCG	64	1065
LHβ-promoter	CTTGATGAGATTGACTTGGTTTTG	CCGTTGCTCAGCAGGCTGTT	58	1178

Supplementary Table S3 | Primer sequences and PCR conditions for real-time PCR for selected gene targets in grass carp

	Forward primer	Reverse primer	Annealing Tm (℃)	Product size (bp)
PrRP1 SP	GCTGCCCACCGCCATCACTC	TCCGCTCTGTCTCTTCCCAAAC	54	238
PrRP2 DP	GTGTGTCTTGCTCTGTCTCCT	CTCTTTGTGCCATCCTTCG	54	173
PrPR-R1a	CTTCCTGCTCCTTTCCTATTT	AGTGGCACGACACCTGTATCA	54	293
PrPR-R1b	CAGCAGCGATGGAGCAAGCAA	CGGGAGGCACAGCGAGAGAGT	54	311
PrPR-R2a	TGGAGTGGACAGATACTACGC	GAATACTGACACGTACACCGT	54	185
PrPR-R2b	TGAGGAGTTCTGGTTGGG	AATGAGGCGGATGTCGAT	54	292
LHβ	GCTCAAAGCTCTTTTCTTCCACCA	GCTGCAGGCTTTCGATGGTACAG	56	319
FSHβ	TTCGTTGTTATGGTGATGCT	CGTGAAAACCGAGTCAGTCC	52	282
GtHα	GATATGACTAACTTTGGATGTG	TAGTAACAGGTGCTACAGTGG	52	263
DRD2	ACTTAAACCCTCCGAGAC	TACACCAAGGACAATAGC	58	296
GnRH2	TGTGTCTAGGTGCCCAGTTTG	GCATCCAGCAGTATTGTCTTCA	60	187
GnRH3	ACTGGTCATACGGTTGGCTTC	CCTCGTCTGTTGGGAAATCTCT	60	202
β-actin	CTGGTATCGTGATGGACTCT	AGCTCATAGCTCTTCTCCAG	56	285

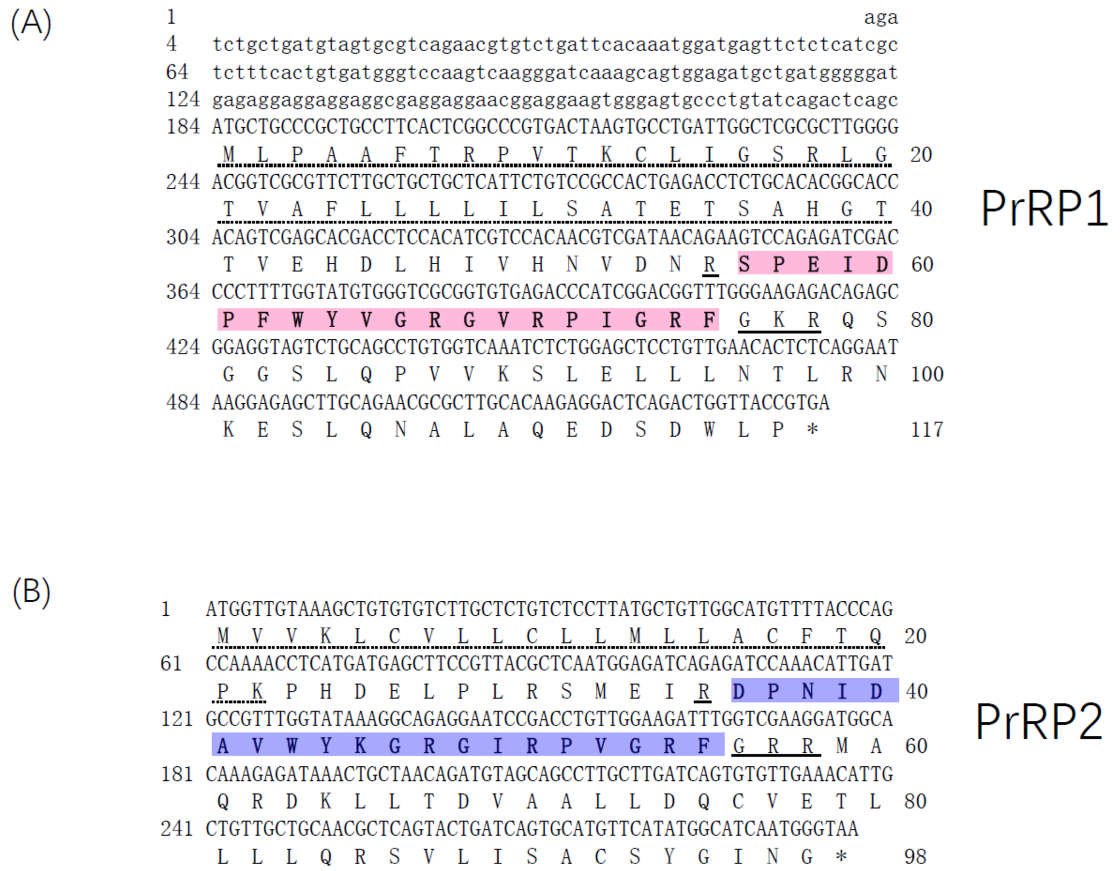

Supplementary Figure S1│Molecular cloning of grass carp PrRP1 & PrRP2. The open reading frame (ORF) of grass carp PrRP1 and PrRP2 cDNA is shown in upper case letters and 5'/3' untranslated region (UTR) sequences in lower case, respectively. The stop codon is marked by an asterisk. Numbering of the deduced amino acid sequences begins with the first methionine of the ORF to the right of each line. Nucleotide numbers are the left of each line. In the corresponding protein sequence, the signal peptide is underlined with a dotted line, the 20-amino acid PrRP1 (SPEIDPFWYVGRGVRPIGRF) and PrRP2 (DPNIDAVWYKGRGIRPVGRF) is boxed in red and blue, respectively. The dibasic cleavage sites (Rand GKR/GRR) flanking the mature peptides are underlined for identification.

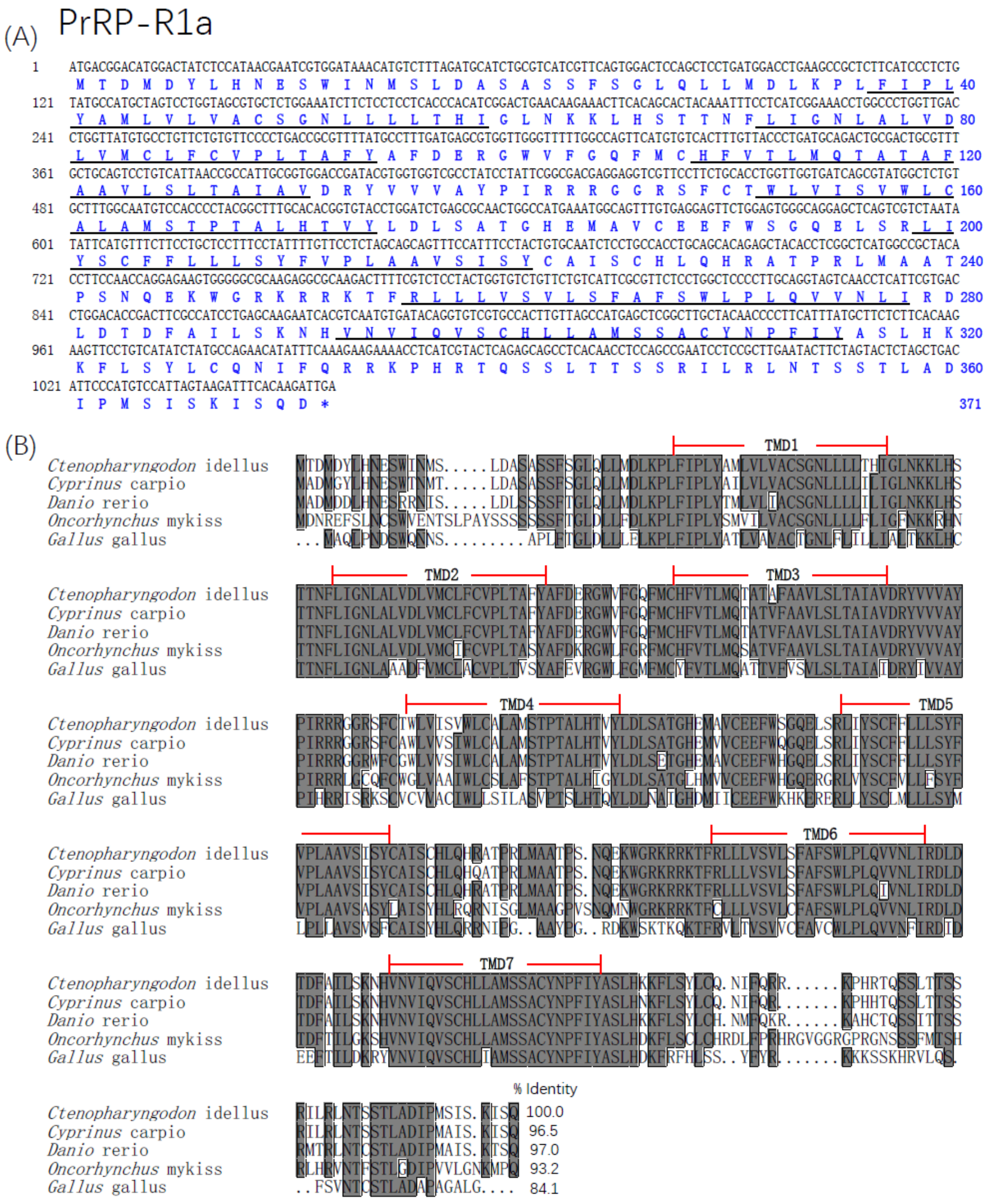

Supplementary Figure S2│Molecular cloning and sequence alignment of grass carp PrRP-R1a. (A) Nucleotide and deduced amino acid sequences of grass carp PrRP-R1a. Numbering of the deduced amino acid sequences begins with the first methionine of the ORF to the right of each line. Nucleotide numbers are to the left of each line. Predicted transmembrane domains (TMD1-7) are underlined. **(B)** Protein sequence alignment of grass carp PrRP-R1a with that of other vertebrates using Clustal-W algorithm with MacVector program. The conserved a.a. residues are boxed in grey. The seven transmembrane domainsare labeled as TMD1-7, respectively.

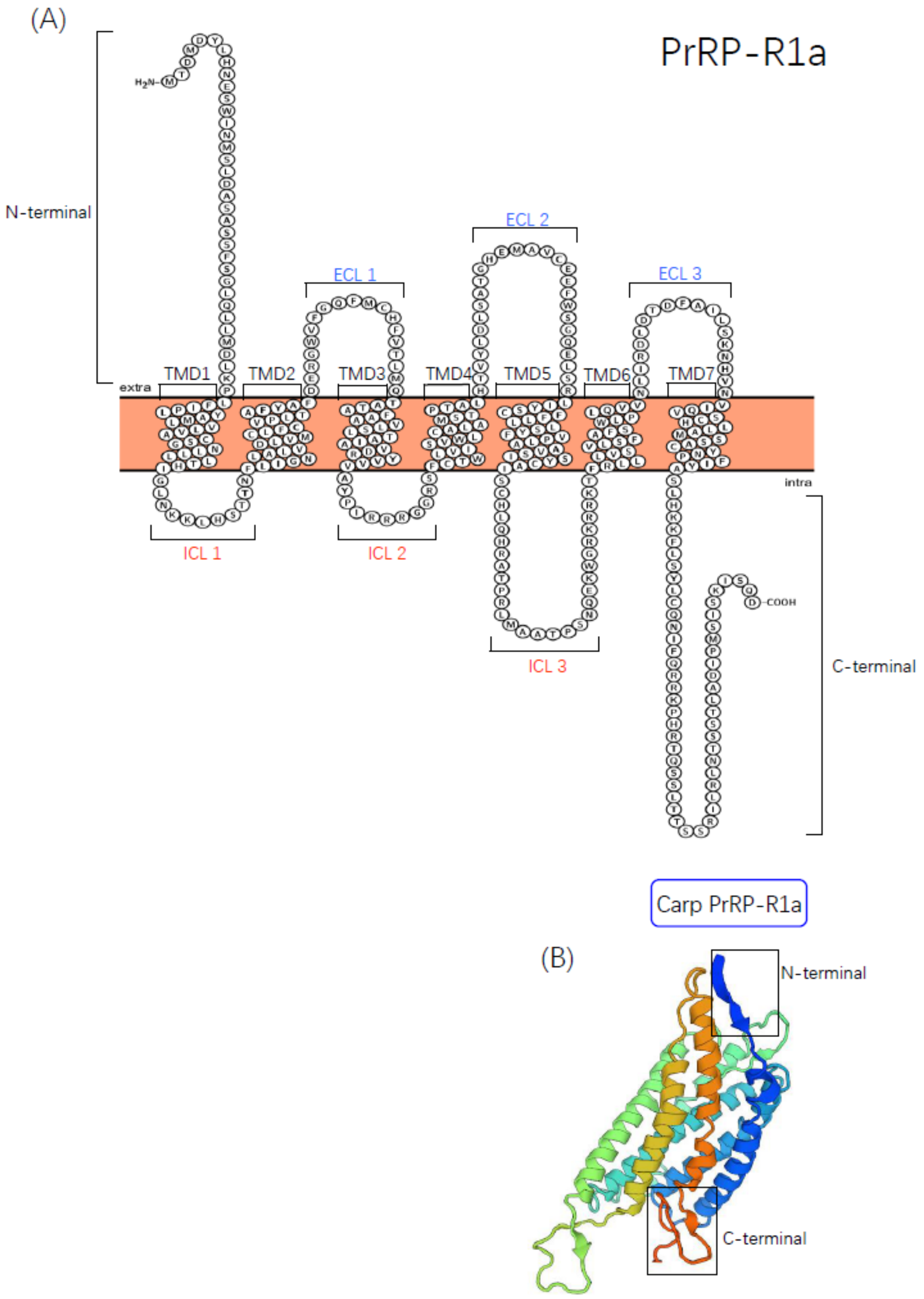

Supplementary Figure S3 | Sequence analysis of grass carp PrRP-R1a. (A) Snake diagram of grass carp PrRP-R1a with Protter program. The seven transmembrane domains, three intracellular domains and three extracellular domains are labeled as TMD1-7, ICL1-3 and ECL1-3, respectively. Sequence identities of transmembrane domains and extracellular domains, intracellular domains, N-termini and C-termini between grass carp and other vertebrates were showed in the Table. **(B)** 3-D protein model of grass carp PrRP-R1a was deduced based on the crystal structure of human PrRP-R1a using SWISS-MODEL program. The location of N-terminal and C-terminal were highlighted, respectively. The amino acids with hydrophobic side chains are colored blue, while those with hydrophilic side chains are colored red.

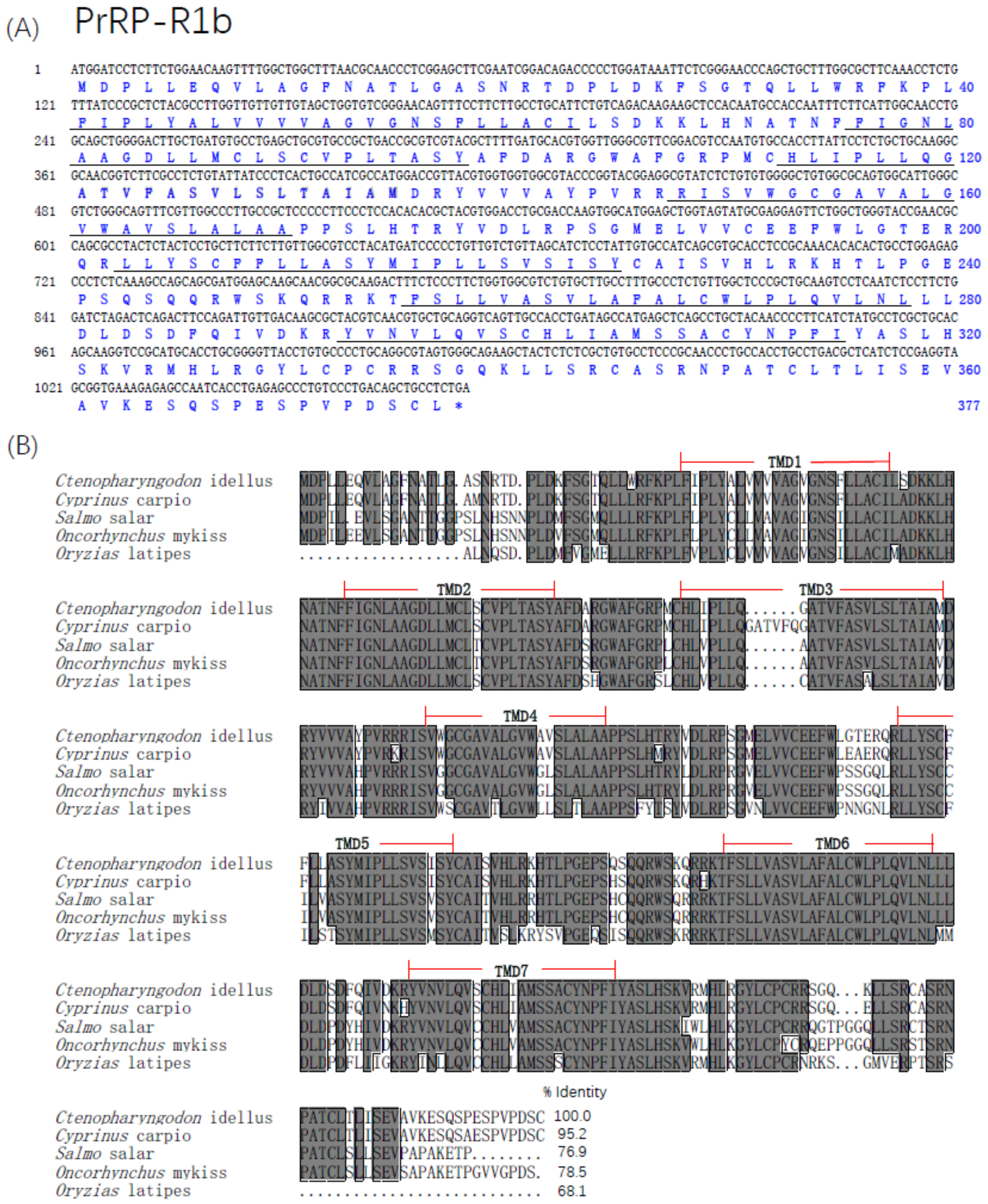

Supplementary Figure S4│Molecular cloning and sequence alignment of grass carp PrRP-R1b. (A) Nucleotide and deduced amino acid sequences of grass carp PrRP-R1b. Numbering of the deduced amino acid sequences begins with the first methionine of the ORF to the right of each line. Nucleotide numbers are to the left of each line. Predicted transmembrane domains (TMD1-7) are underlined. **(B)** Protein sequence alignment of grass carp PrRP-R1b with that of other vertebrates using Clustal-W algorithm with MacVector program. The conserved a.a. residues are boxed in grey. The seven transmembrane domainsare labeled as TMD1-7, respectively.

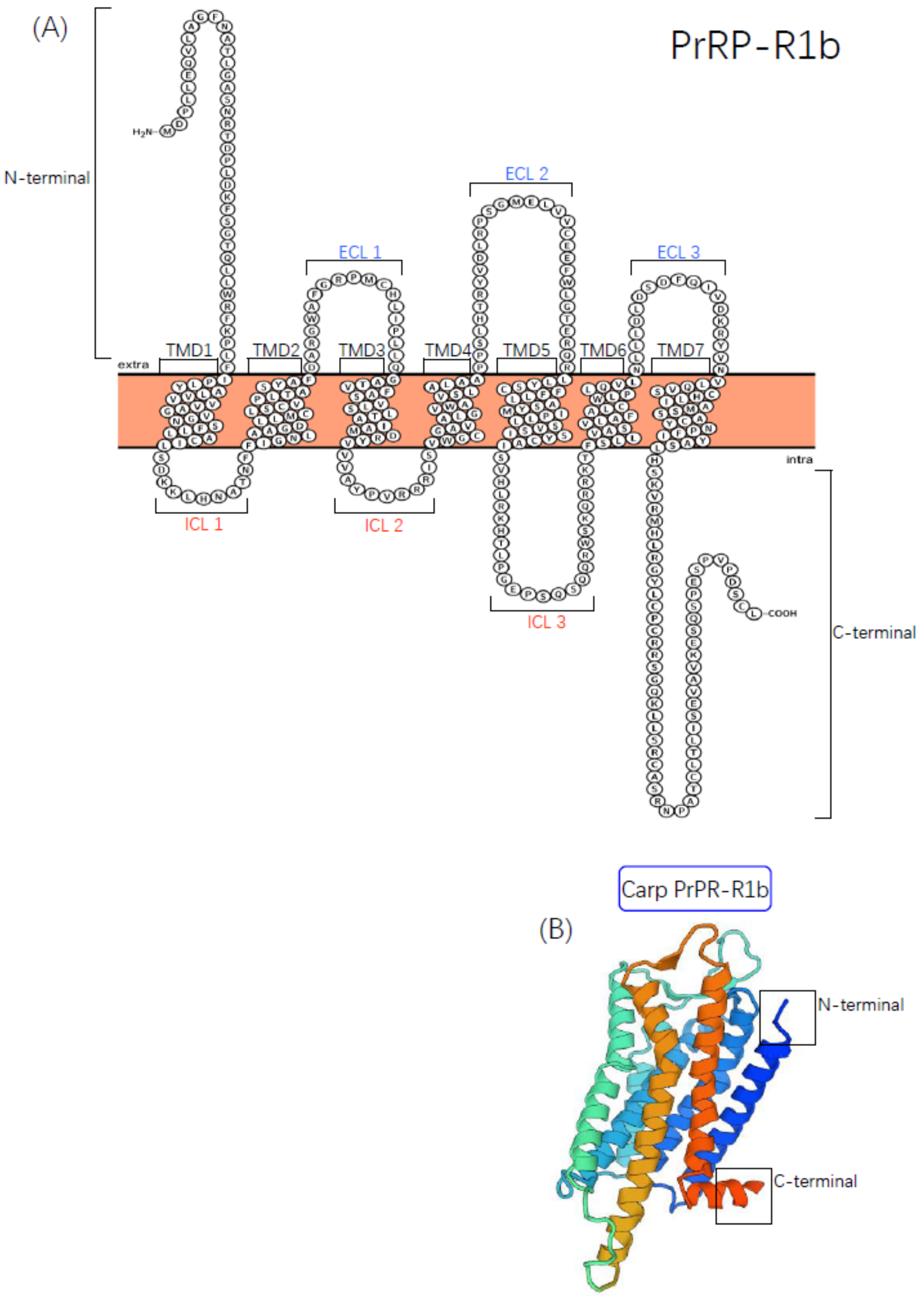

Supplementary Figure S5│Sequence analysis of grass carp PrRP-R1b. (A) Snake diagram of grass carp PrRP-R1b with Protter program. The seven transmembrane domains, three intracellular domains and three extracellular domains are labeled as TMD1-7, ICL1-3 and ECL1-3, respectively. Sequence identities of transmembrane domains and extracellular domains, intracellular domains, N-termini and C-termini between grass carp and other vertebrates were showed in the Table. **(B)** 3-D protein model of grass carp PrRP-R1b was deduced based on the crystal structure of human PrRP-R1b using SWISS-MODEL program. The location of N-terminal and C-terminal were highlighted, respectively. The amino acids with hydrophobic side chains are colored blue, while those with hydrophilic side chains are colored red.

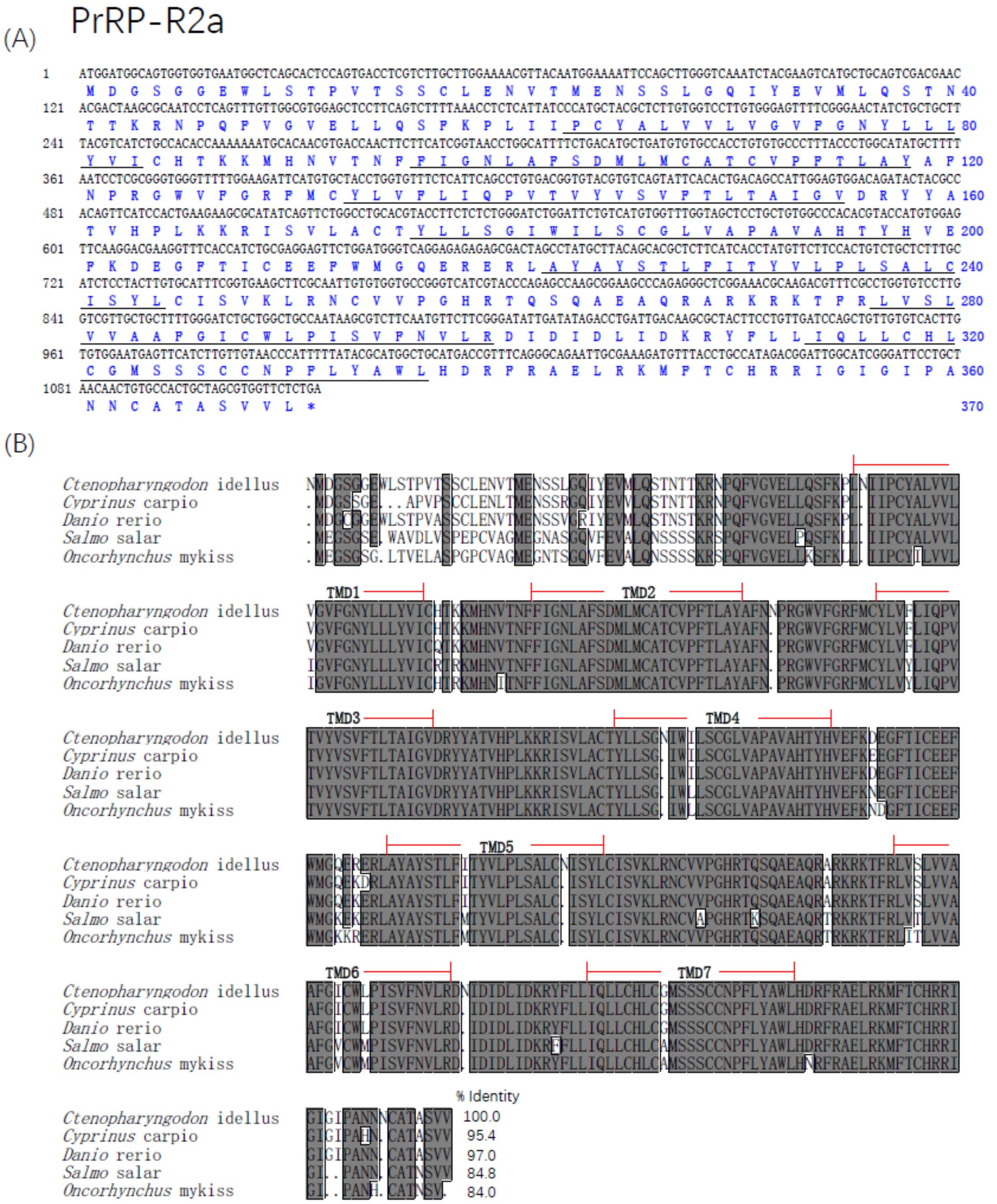

Supplementary Figure S6│Molecular cloning and sequence alignment of grass carp PrRP-R2. (A) Nucleotide and deduced amino acid sequences of grass carp PrRP-R2. Numbering of the deduced amino acid sequences begins with the first methionine of the ORF to the right of each line. Nucleotide numbers are to the left of each line. Predicted transmembrane domains (TMD1-7) are underlined. **(B)** Protein sequence alignment of grass carp PrRP-R2 with that of other vertebrates using Clustal-W algorithm with MacVector program. The conserved a.a. residues are boxed in grey. The seven transmembrane domainsare labeled as TMD1-7, respectively.

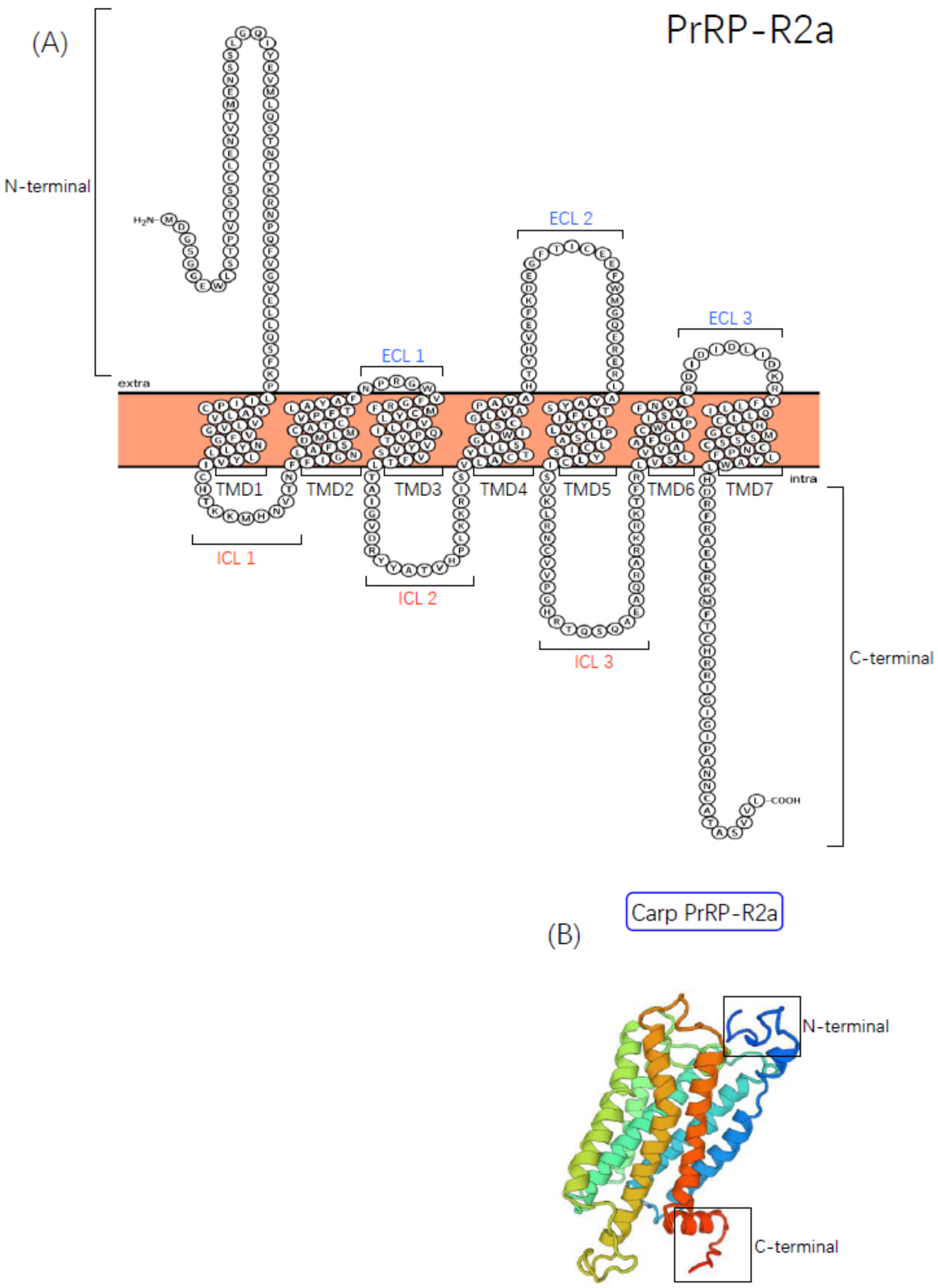

Supplementary Figure S7 | Sequence analysis of grass carp PrRP-R2a. (A) Snake diagram of grass carp PrRP-R2a with Protter program. The seven transmembrane domains, three intracellular domains and three extracellular domains are labeled as TMD 1-7, ICL1-3 and ECL1-3, respectively. Sequence identities of transmembrane domains and extracellular domains, intracellular domains, N-termini and C-termini between grass carp and other vertebrates were showed in the Table. **(B)** 3-D protein model of grass carp PrRP-R2a was deduced based on the crystal structure of human PrRP-R2a using SWISS-MODEL program. The location of N-terminal and C-terminal were highlighted, respectively. The amino acids with hydrophobic side chains are colored blue, while those with hydrophilic side chains are colored red.

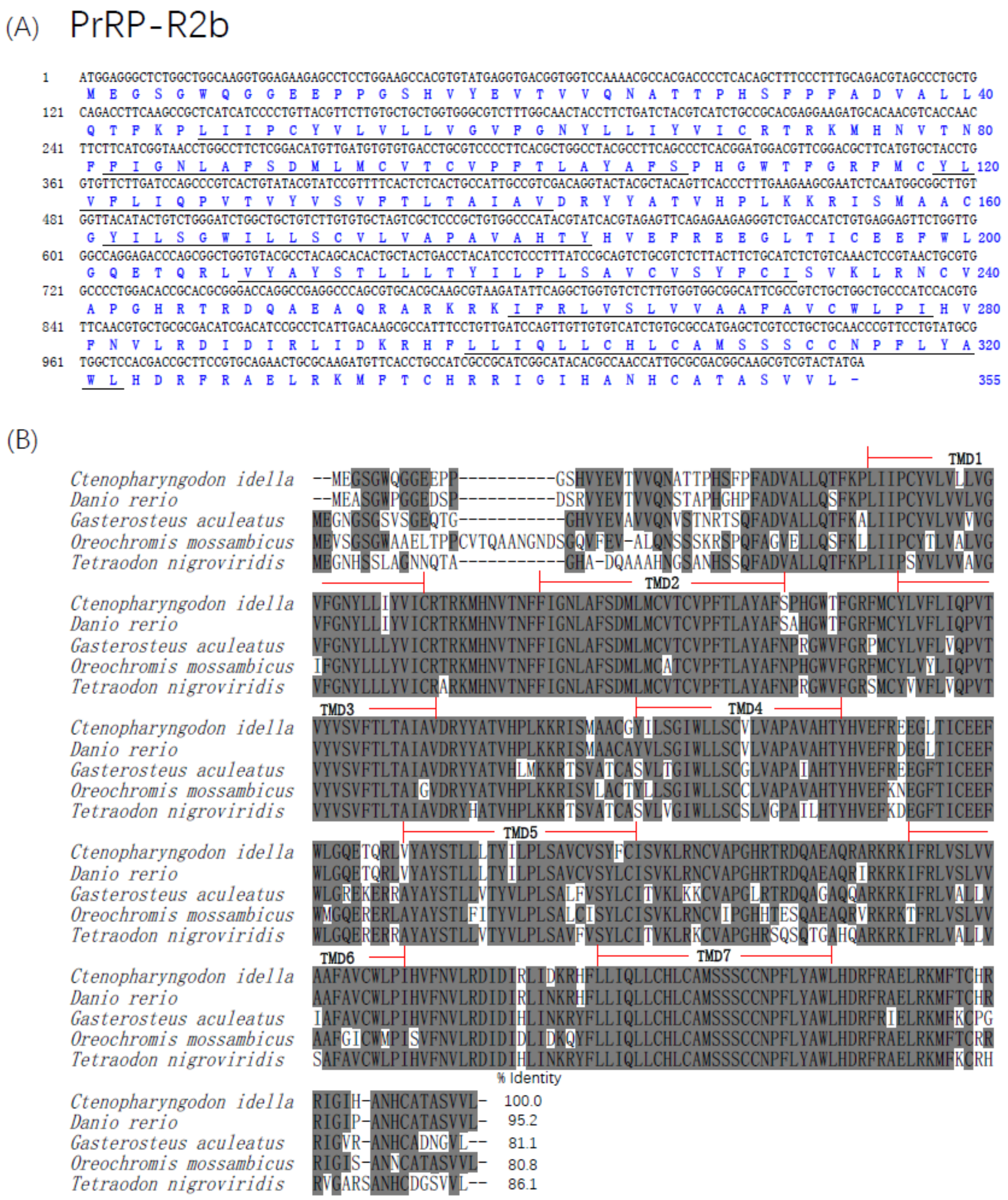

Supplementary Figure S8│Molecular cloning and sequence alignment of grass carp PrRP-R2b. (A) Nucleotide and deduced amino acid sequences of grass carp PrRP-R2b. Numbering of the deduced amino acid sequences begins with the first methionine of the ORF to the right of each line. Nucleotide numbers are to the left of each line. Predicted transmembrane domains (TMD1-7) are underlined. **(B)** Protein sequence alignment of grass carp PrRP-R1b with that of other vertebrates using Clustal-W algorithm with MacVector program. The conserved a.a. residues are boxed in grey. The seven transmembrane domainsare labeled as TMD1-7, respectively.

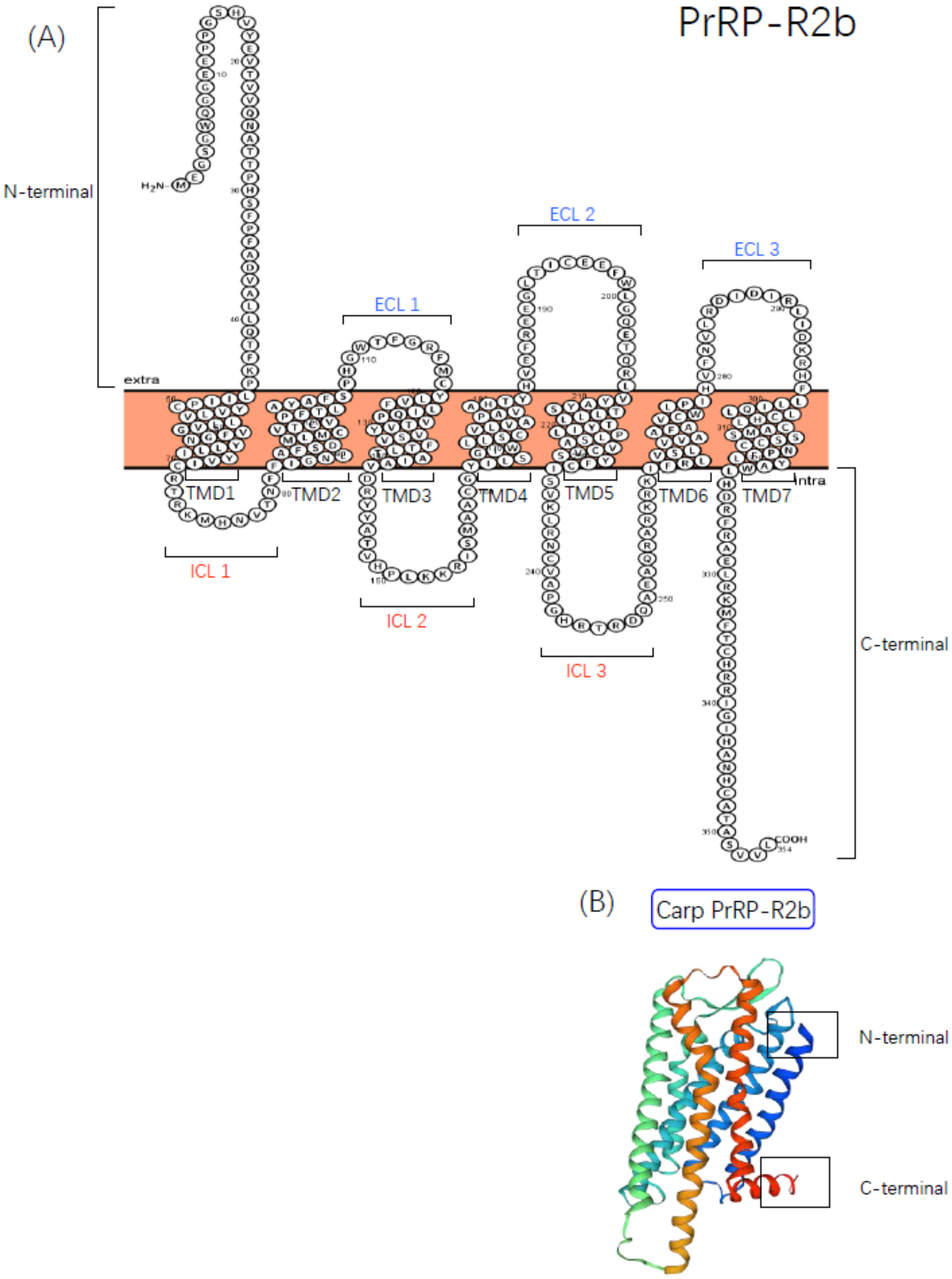

Supplementary Figure S9│**Sequence analysis of grass carp PrRP-R2b.** **(A)** Snake diagram of grass carp PrRP-R2b with Protter program. The seven transmembrane domains, three intracellular domains and three extracellular domains are labeled as TMD 1-7, ICL1-3 and ECL1-3, respectively. Sequence identities of transmembrane domains and extracellular domains, intracellular domains, N-termini and C-termini between grass carp and other vertebrates were showed in the Table. **(B)** 3-D protein model of grass carp PrRP-R2b was deduced based on the crystal structure of human PrRP-R2b using SWISS-MODEL program. The location of N-terminal and C-terminal were highlighted, respectively. The amino acids with hydrophobic side chains are colored blue, while those with hydrophilic side chains are colored red.

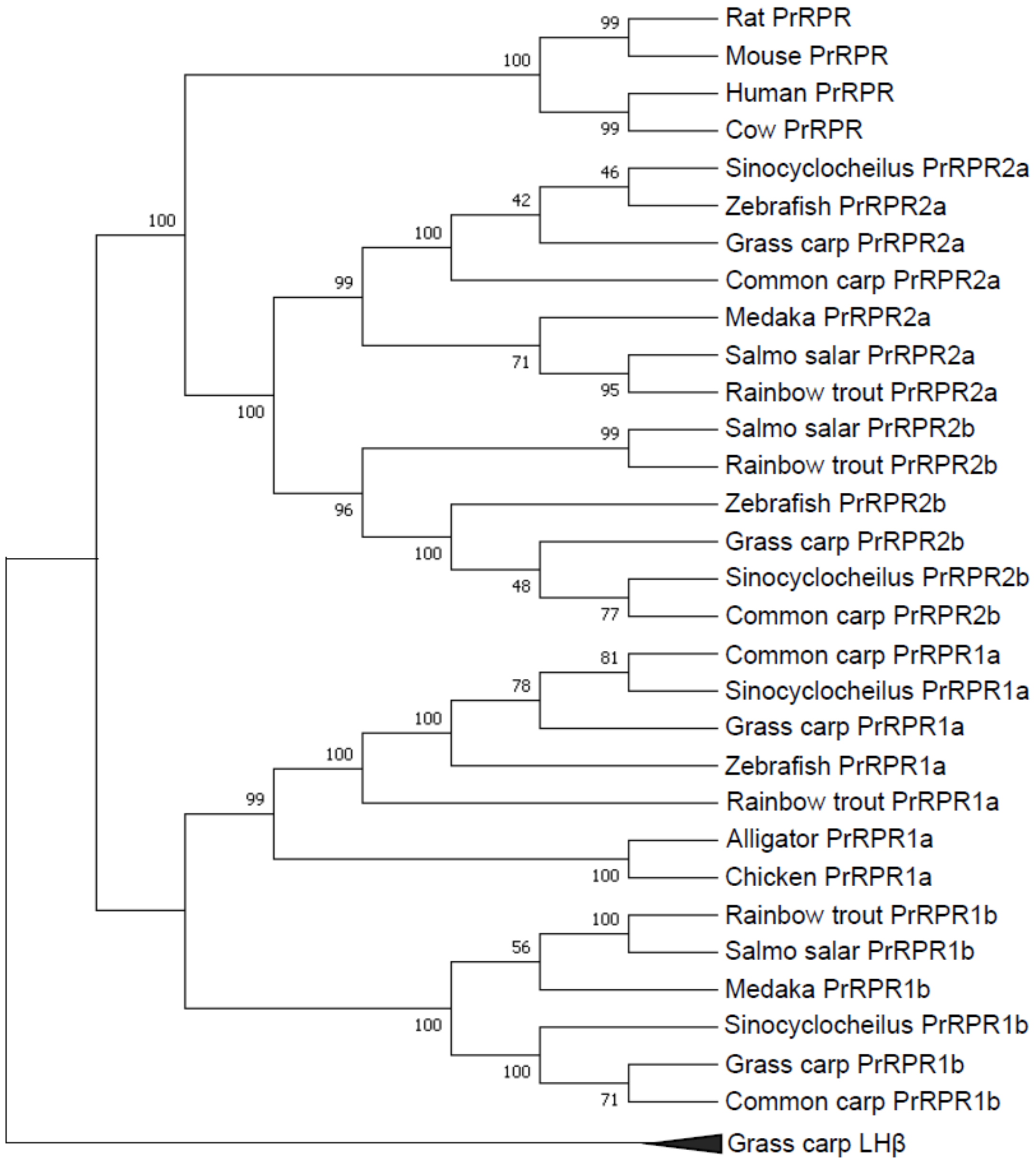

Supplementary Figure S10│Phylogenetic analysis of vertebrate PrPR amino acid sequences using neighbor-joining method with MEGA X. The grass carp PrPR1a, PrPR1b, PrPR2a and PrRP2b were obtained and used to cluster into four branches. The numbers presented in the guide tree are the percentage of bootstrap values based on 1000 bootstrap. The other PrRP-Rs were collected form NCBI database.

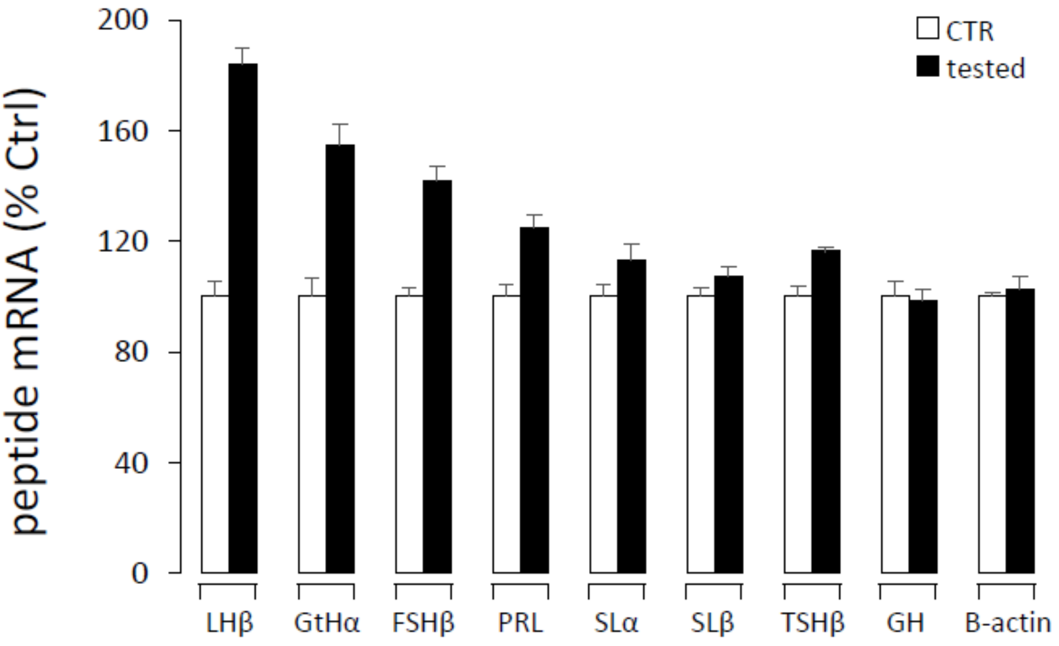

Supplementary Figure S11│The effect induced by PrRPs on LHβ, GtHα, FSHβ, PRL, SLα, SLβ, TSHβ, GH mRNA expression in grass carp pituitary cells. Preliminary experiment was performed that primary cultured pituitary cells were initially treated with PrRP1 (the final concentration 1 μM) for 24 h. After drug treatment, the pituitary cells were extracted to total RNA, reverse transcription and used for RT-PCR to detect the target gene expression level. Besides, the expression level of β-actin was considered as an internal control.

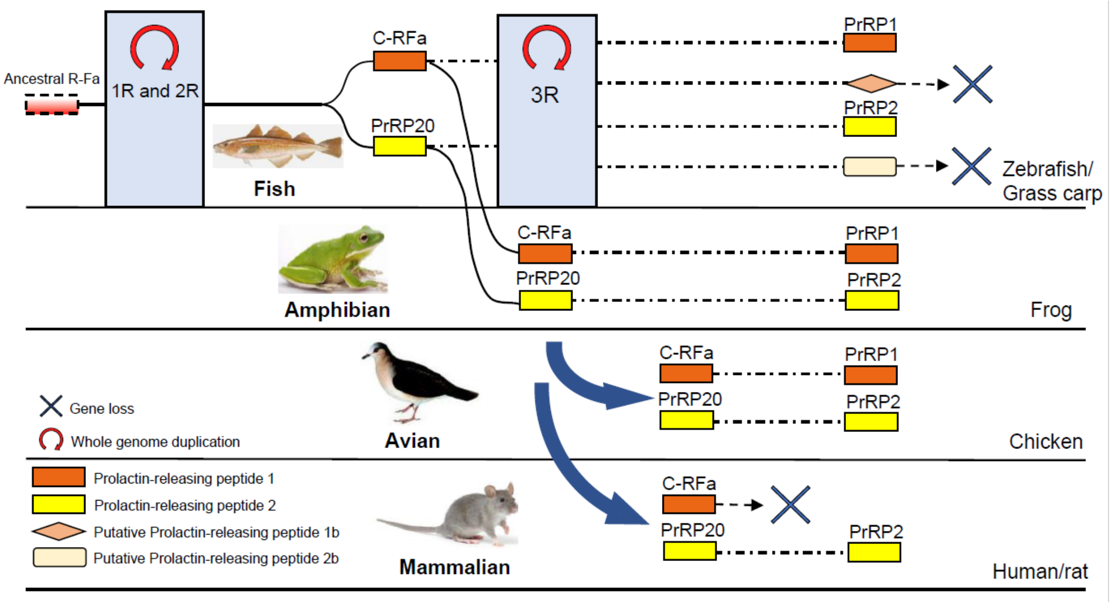

Supplementary Figure S12│A speculative evolutionary process of PrRPs with respect to several rounds of genome duplication. The peptides and gene are shown by labelling in the key. The genome duplication events are noted by light-blue boxes. The evolutionary path is marked with arrows.

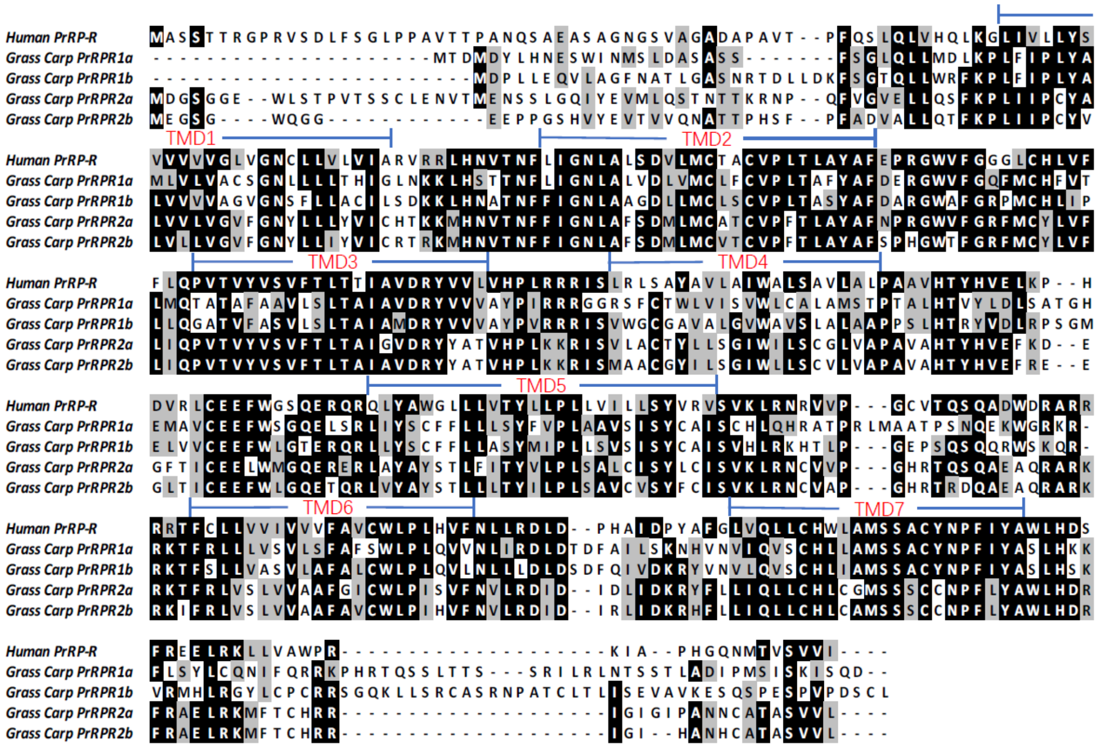

Figure S13. Sequence alignment of grass carp PrRP-R1a, PrRP-R1b, PrRP-R2a, PrRP-R2b and human PrRP-R. Protein sequence alignment of grass carp PrRP-R1a, PrRP-R1b, PrRP-R2a, PrRP-R2b and human PrRP-R using Clustal-W algorithm with MacVector program. The lower conserved a.a. residues are boxed in grey where higher are in black. The seven transmembrane domainsare labeled as TMD1-7, respectively.

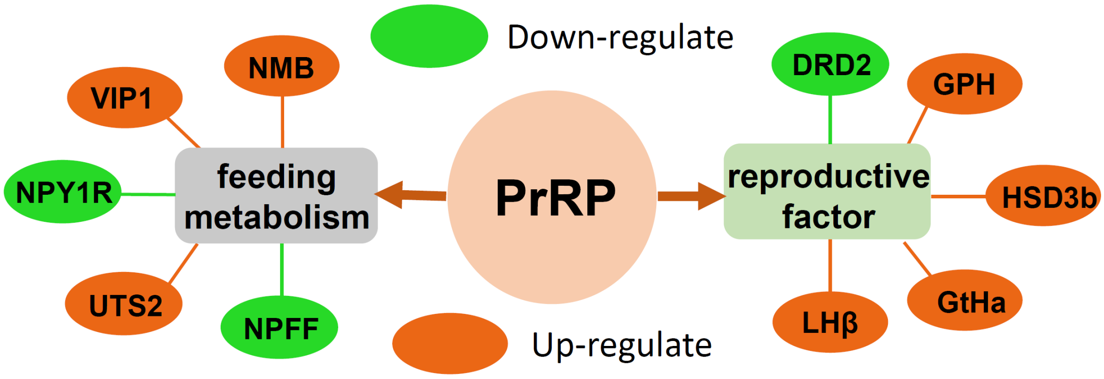

Figure S14. **Selected target genes by High-throughput RNA-seq.** Using High-throughput RNA-seq data, we intentionally selected aiming genes which were considered existing connection with feeding metabolism and reproductive factor, and were marked with green indicating PrRP down-regulate, meanwhile red represents up-regulate.
